# Updates and Original Case Studies Focused on the NMR-Linked Metabolomics Analysis of Human Oral Fluids Part III: Implementations for the Diagnosis of Non-Cancerous Disorders, Both Oral and Systemic

**DOI:** 10.3390/metabo13010066

**Published:** 2023-01-01

**Authors:** Martin Grootveld, Georgina Page, Mohammed Bhogadia, Kayleigh Hunwin, Mark Edgar

**Affiliations:** Leicester School of Pharmacy, De Montfort University, The Gateway, Leicester LE1 9BH, UK

**Keywords:** saliva, metabolomics, ^1^H NMR analysis, NMR-based metabolomics, oral diseases, periodontal diseases, dental caries, systemic diseases, acute sore throat conditions (pharyngitis), diagnosis, prognostic monitoring, chemical pathology

## Abstract

This communication represents Part III of our series of reports based on the applications of human saliva as a useful and conveniently collectable medium for the discovery, identification and monitoring of biomarkers, which are of some merit for the diagnosis of human diseases. Such biomarkers, or others reflecting the dysfunction of specific disease-associated metabolic pathways, may also be employed for the prognostic pathological tracking of these diseases. Part I of this series set the experimental and logistical groundwork for this report, and the preceding paper, Part II, featured the applications of newly developed metabolomics technologies to the diagnosis and severity grading of human cancer conditions, both oral and systemic. Clearly, there are many benefits, both scientific and economic, associated with the donation of human saliva samples (usually as whole mouth saliva) from humans consenting to and participating in investigations focused on the discovery of biomolecular markers of diseases. These include usually non-invasive collection protocols, relatively low cost when compared against blood sample collection, and no requirement for clinical supervision during collection episodes. This paper is centred on the employment and value of ‘state-of-the-art’ metabolomics technologies to the diagnosis and prognosis of a wide range of non-cancerous human diseases. Firstly, these include common oral diseases such as periodontal diseases (from type 1 (gingivitis) to type 4 (advanced periodontitis)), and dental caries. Secondly, a wide range of extra-oral (systemic) conditions are covered, most notably diabetes types 1 and 2, cardiovascular and neurological diseases, and Sjögren’s syndrome, along with a series of viral infections, e.g., pharyngitis, influenza, HIV and COVID-19. Since the authors’ major research interests lie in the area of the principles and applications of NMR-linked metabolomics techniques, many, but not all, of the studies reviewed were conducted using these technologies, with special attention being given to recommended protocols for their operation and management, for example, satisfactory experimental model designs; sample collection and laboratory processing techniques; the selection of sample-specific NMR pulse sequences for saliva analysis; and strategies available for the confirmation of resonance assignments for both endogenous and exogenous molecules in this biofluid. This article also features an original case study, which is focussed on the use of NMR-based salivary metabolomics techniques to provide some key biomarkers for the diagnosis of pharyngitis, and an example of how to ‘police’ such studies and to recognise participants who perceive that they actually have this disorder but do not from their metabolic profiles and multivariate analysis pattern-based clusterings. The biochemical and clinical significance of these multidimensional metabolomics investigations are discussed in detail.

## 1. Introduction

This report represents Part III of our detailed Commentary centred on metabolomics investigations of human saliva. Part I of this series specifically evaluated and extensively reviewed the scientific development, applications and contemporary employment of NMR-linked analysis strategies for exploring the identification, quantification, the molecular nature, metabolic and physiological sources, along with the dispositions of a very wide range of salivary biomolecules simultaneously [1]. A range of important considerations, one of the most important being the instigation of sufficient abstention periods from oral activities prior to sample collection, were critically evaluated and further explored. Indeed, we found that converse to many claims that a minimum of a 1 h or so abstention period is sufficient for this purpose, an absolute minimum of 2–4 h was found to be preferential, largely because dietary constituents such as sucrose and glucose, and other endogenous agents such as citrate, remain ^1^H NMR-detectable in whole mouth salivary supernatant samples (WMSSs) up to and beyond these time-points following food consumption regimens. No doubt, the employment of more sensitive analytical techniques will serve to prove that perhaps this 2–4 h restriction should be further extended, perhaps up to or even longer than 8–12 h, a period which our group commonly employs for such investigations.

Moreover, from factor analysis performed on a very large number of healthy control participants, a further unique finding was the segregation of metabolite ‘predictor’ variables into four orthogonal components, the most important being those ascribable to patterns of biomolecules arising from either host or oral microbiome sources, the latter containing highly significant loadings from some common bacterial salivary catabolites such as propionate, *n*-caproate, *n*- and *iso*-butyrates, and 5-aminopentanoate, etc. Although further detailed investigations may be required, this model appeared to provide us with valuable information regarding the distinction of participants with an oral microbiome-dominant pattern of metabolites from those with a host-dominant one, and those with an approximately equivalent admixture of these two major salivary metabolite origins. Indeed, the relevant factor analysis scores vectors of WMSS samples on differential orthogonal principal components (PCs) collected from these control human participants provided sufficient information to permit these distinctions.

Areas also reviewed included: (1) methods available for the critical passage, storage and preparation of oral fluids, notably WMS for NMR analysis, the latter including the addition of salivary metabolite-sustaining microbicidal agents; (2) the best choice pulse sequences for spectral acquisition, and the many assignment advantages offered by the implementation of two-dimensional (2D) NMR techniques; and (3) the preprocessing of spectral profiles, specifically the chemical shift bucketing of ^1^H NMR resonances, along with metabolite quantification, and appropriate protocols for experimental multivariate (MV) dataset normalisation, transformation and scaling strategies.

This expansive Commentary paper was then followed-up in Part II of this series [2], which was largely focused on the applications, operation, validation, reliability and pathological extensions and implications of ‘state-of-the-art’ ^1^H NMR technologies to the diagnosis and prognostic tracking of human cancers, both oral and systemic. The extrapolation of results from these studies to the identification of dysfunctions or imbalances in metabolic pathways was also reviewed, as was the further application of these strategies to the pinpointing of drug targets, and the consequent design and development of suitable drug therapies for the diseases specified. Additionally, included was a unique case study which explored the nature and levels of resonances contributing towards the acute-phase protein (APP) acetamido-CH_3_ function region of the ^1^H NMR profiles of WMSS samples (δ = 1.80–2.20 ppm); their relevance to the monitoring of inflammatory diseases and cancers; and most importantly, the identification of interferants also with signals within this region, a development which may preclude its diagnostic applications in this biofluid and perhaps other biofluids too.

As a logical and planned extension of this work, here, in Part III, the authors explore the applications of these salivary biomolecules, or specific patterns of them, as potential biomarkers for non-cancerous human diseases, including oral, oral-systemic and systemic.

Currently, blood-derived biofluids, specifically serum and plasma, are unanimously the most frequent and widely accepted choice for clinical laboratory biomarker evaluations, largely because blood represents the circulating fluid that envelopes and hence has ready access to all tissues and organs, and in principle it may acquire metabolic products generated in diseased environments, such as tumours. Therefore, up- or downregulations in the levels of specific low-molecular-mass, protein or other types of biomarkers present in blood plasma or serum have been linked to the induction, development and progression of human diseases, a process which can lead to now generally accepted clinical applications. However, WMS, or more specifically its supernatant (WMSS) prepared from the removal of cells, microbes and debris via centrifugation, represents an appealing biofluid for disease biomarker discovery and diagnosis, most especially because of its ease of collection from human participants.

For situations where salivary biomolecules or biomarkers directly arise from blood (host) contributions, concentrations of these agents, and those of immunological agents determined in WMSS samples, may correlate strongly with corresponding blood levels, although it is generally accepted that in the majority of cases, salivary levels of such agents are considerably lower than those present in blood plasma [1,3]. Therefore, the replacement of blood serum or plasma samples with saliva for the analysis of biomarkers remains of much interest since its sample collection is much less invasive and intrusive, and does not present any of the recorded, albeit low risks associated with whole blood sample collection, for example the risk of feinting in a small proportion of donors (outlined in detail in Ref. [1]).

In this follow-up paper (Part III), the authors have performed rigorous reviews of reported results obtained from a wide range of investigations conducted to determine the value of mainly NMR-based salivary metabolomics studies for the diagnosis and/or prognostic monitoring of both oral and non-oral (systemic) diseases. Problems commonly experienced with these studies include those involving the timings of saliva sample collections, sample transport and preparation regimens, and the uniformity and future homogenization of techniques for the NMR analysis of this biofluid, for example pulse sequences utilized (including those for intense H_2_O solvent and less intense macromolecule resonance suppression, for example), internal standards and quantification protocols employed, etc. An important further issue is the intermittent misassignment of NMR resonances, ^1^H or otherwise, by some researchers.

Following this Section, Section 2 commences with an update on the scientific and clinical benefits presented by datasets acquired from salivary metabolomics studies, and more detailed considerations of saliva sample collection and their laboratory processing, together with those concerning problems encountered with the ^1^H NMR analysis of biomolecules containing exchangeable ^1^H nuclei. This Section also features a review of the possible advantages and conveniences offered by the metabolic probing of an alternative analytical matrix (mouth rinsed water washouts) rather than WMSSs themselves; differences between the metabolic profiles of different types of human saliva samples are also delineated. Subsequently, Section 3 covers the appliance of these experimental regimens towards the diagnosis and prognostic monitoring of human oral diseases (mainly gingivitis and more severe periodontal diseases (PDs), and dental caries), whereas Section 4 involves the employment of human saliva for the identification, detection and perhaps future monitoring of a variety of extra-oral (systemic) diseases, notably type 1 and 2 diabetes, cardiovascular conditions, Sjörgen’s syndrome, neurological and respiratory diseases, chronic apical abscess, and selected viral infections, the latter including pharyngitis (acute sore throat conditions), HIV, and inevitably COVID-19 infections. Inherent limitations associated with salivary metabolomics investigations are adequately outlined and discussed in Parts I and II of this series of publications (Refs. [1,2], respectively). Diseases which, to date, have not yet been subjected to NMR-based studies, but instead have utilised other types of bioanalytical techniques for MV metabolomics investigations, are also included. For the diseases specified, the possibilities and potential advantages arising from employment of NMR-linked approaches are briefly discussed. An additional input features a case study (Section 5), which involves an ^1^H NMR-linked metabolomics investigation of acute sore throat disease in humans, notably its possible diagnosis from the salivary metabolic profiles of sample donors and associated complications, and the implications of results acquired therefrom regarding viral-induced disturbances to host metabolic pathways. This is then followed by a Section dedicated to diagnostic metabolomics investigations involving the analysis of saliva specimens collected from animals (Section 6). Subsequently, we include a discussion section encompassing deliberations of methods for the validation of salivary biomarkers, potential clinical diagnoses and disease time-course evaluations, and in view of prospective differences between participants with differing ethnicities, diets and geographic locations, the prospective global applications of the salivary metabolomics techniques (Section 7). Finally, a generalized Concluding Remarks section (Section 8) is provided, which includes possible limitations of metabolomics study experimental designs and performances, together with the experimental reliabilities of results acquired.

## 2. Scientific and Clinical Benefits Offered by Salivary Metabolomics Investigations

### 2.1. Further Recent Developments on the Advantages, Collection, Laboratory Processing, Storage, Spectral/Data Acquisition, Data Analysis and ‘Policing’ of Salivary Metabolomics Investigations Using ^1^H NMR Analysis and Other Techniques

In 2010, Henson and Wong [4] surmised that saliva acts as an ideal ‘translational’ research probe and diagnostic medium for the provision of molecular biomarkers for a now very wide range of systemic as well as oral diseases. Indeed, a robust ability to analyse this biofluid for the purpose of monitoring health status, in addition to various diseases, is currently an important summit to traverse for the advancement of human health research avenues. Hence, to date, this biofluid has been employed to detect risks or perform disease status monitoring for dental caries, periodontitis, salivary gland disorders, oral and systemic cancers, together with viral diseases, e.g., HIV and hepatitis C (HCV), amongst others. These researchers also reviewed methods for optimising the donation, collection, storage and processing of saliva samples in order to explore downstream molecular developments and applications. Indeed, previously unrecognised biotechnological advancements have permitted such investigations to be conducted on high-throughput scales, processes which have undoubtedly given rise to some breakthrough discoveries regarding biomarkers for human diseases, particularly. those for diagnostic and prognostic tracking purposes.

Of notable interest, the combination of datasets from differential biofluid types for single patients using two or more bioanalytical strategies could, in principle, significantly broaden coverage of the salivary metabolome. Using this approach, Martias et al. [5] analysed human urine, blood and faeces, in addition to saliva samples using expansive analytical platforms, specifically reverse-phase-ultra-high-pressure liquid-chromatography mass-spectrometric (RP-UHPLC-MS), and hydrophilic interaction liquid-chromatography-ultra-high performance liquid chromatography-mass spectrometric (HILIC-UHPLC-MS) techniques, in addition to ^1^H NMR analysis. Accordingly, each class of sample was prepared by a unique preparation process for such multi-platform analysis, although it is our view that such a strategy would be quite onerous and labour-intensive. The researchers involved assessed this multi-platform methodology for its bioanalytical ebullience and validity, and concluded that it was able to produce a representative metabolic ‘map’.

The review presented by Boroumand et al. [6] firstly briefly outlines the differential inputs of metabolite sources to WMS samples, and this is followed by a description of ‘dos and don’ts’ for the collection, laboratory preparation and storage of such specimens; our group have also recently covered all these issues in Ref. [1]. A further section of this paper explores the current use of WMS for the provision of valuable biomolecular diagnostic information, and its employment for the distinction of both local and systemic diseases. Additionally, provided are descriptions of the future benefits offered by some rather unconventional techniques for such ‘omics’ studies. Moreover, this review furnishes us with some complications which have to be considered to circumvent false positive and negative criteria, and these included the sufficiency of the original experimental design and the number of participant sample donors recruited, together with the correct or acceptable selection of a control group. These authors also note the application of this biofluid and bioanalytical techniques to the detection and quantification of drugs, either therapeutic or abused, again studies that our group have previously conducted [7].

**Figure 1:**
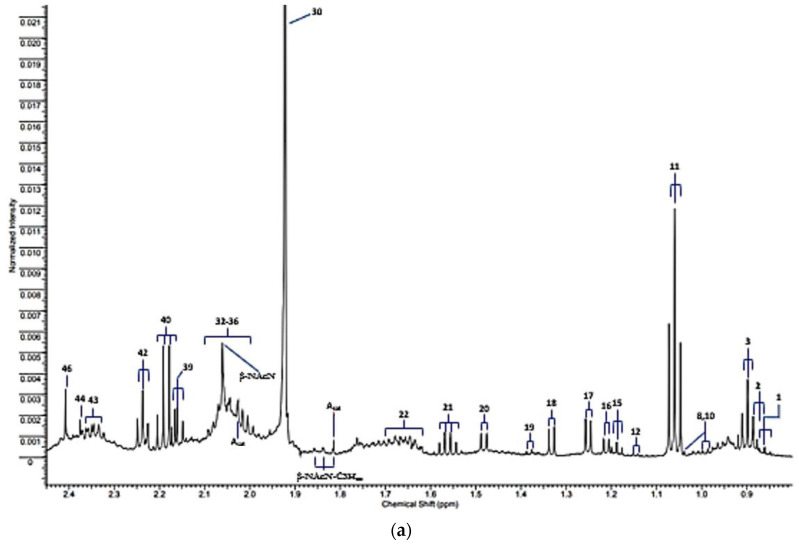

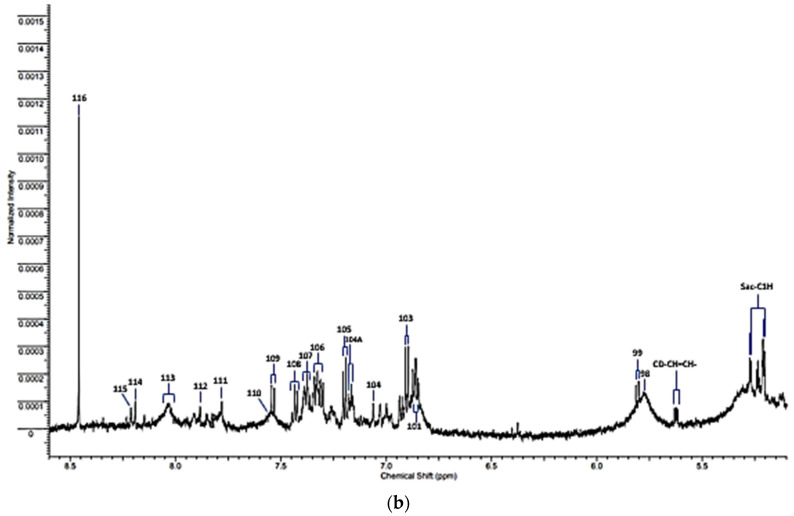
Partial ^1^H NMR profiles of a WMSS sample provided by a healthy human participant, stored and prepared for analysis according to our protocols described in [1]. (**a**,**b**) 0.80–2.45 and 5.10–8.60 ppm regions of spectra acquired, respectively. Typical spectra are shown. Abbreviations: Assignment labels correspond to those available in Table 1; some tentative assignments are also indicated, as are unassigned signals. Single-pulse *noesy-presat* ^1^H NMR spectra were recorded on a Bruker Avance-600 spectrometer operating at frequency of 600.13 MHz and a probe temperature of 293 K. The highly intense H_2_O/HOD resonance (δ = 4.80 ppm) was subdued with radiofrequency pulse presaturation. Pulsing conditions were: sweep width 8389 Hz; 5 µs pulses; 1.0 s pulse repetition rate; 32,768 (then zero-filled to 65,536) datapoints; 128 transients. An exponential line-broadening function of 0.30 Hz was applied to FIDs before Fourier transformation.

The expanded 0.80–2.45 and 5.10–8.60 ppm regions of a typical 600 MHz *noesy-presat* ^1^H NMR spectrum of a WMSS sample collected from a healthy human participant in our laboratories according to our experimental protocol [1] are shown in Figure 1a,b, respectively. Full spectral assignments for these partial profiles are provided in Table 1, although it should be noted that a total of 50 or so additional assigned resonances are visible within the 2.45–5.10 and >8.60 ppm regions of spectra acquired, and readers are referred to Refs. [1,2] to view/review these.

#### 2.1.1. Updated Treatise on Saliva Sample Collection, Laboratory Processing and Storage

Part I of this series of three publications [1] already outlines full details regarding sample collection, processing and storage in a lengthy Section 6 of that paper, which is entitled ‘Recommended Protocols for the Collection, Storage and Preparation of Human Saliva Specimens for NMR Analysis: Precautions Against Artefacts, and Artefactual Metabolite Generation or Consumption’. This section of that paper includes sub-sections focussed on saliva sample collection (Part I, Section 6.1); abstention from oral activities prior to saliva sample donation: How long is really necessary? (Part I, Section 6.2); the post-collection maintenance of salivary biomolecular profiles: Preparation, transport and storage of saliva samples for NMR analysis (Part I, Section 6.3); and reported developments of recommended protocols for saliva sample collection, preparation and storage (Part I, Section 6.4). Readers are therefore referred to this report to acquire further relevant details on this important topic.

In order for salivary metabolomics investigations to be successful, the overall integrity of saliva samples (both whole and supernatants derived therefrom), and the conservation of levels of salivary biomolecules so that they remain identical to those present at the point-of-collection, continues to be a consideration of critical importance. Hence, sufficient precautions should be taken to circumvent or limit artefactual chemical changes to pre-collected WMSS samples from this point until ready for spectral analysis, most notably during sample preparation and any storage periods involved. In view of these contemplations, and to provide reliable protocols for all future prospective NMR-based salivary metabolomics investigations, it is of much importance to establish recommended methods and strategies for these sample collection processes. Clearly, such developments will serve to nurture bioanalytical validity, and allow direct comparisons between comparable or related studies conducted by other ‘omics’ research laboratories. Therefore, these protocols will serve to tackle any issues arising from differing sample collection regimens, along with problems arising from non-identical sample preparation, storage and NMR acquisition frameworks. Interestingly, Gardner et al. [8] conducted a very work-intensive review study of these sample collection, preparation and storage techniques, and found that at least some already available protocols for salivary ^1^H NMR analysis involving the employment of unbuffered internal TSP as a quantitative reference standard (as well as a chemical shift (δ) parameter reference), were adequate for quantitative NMR (QNMR) determinations made on the majority of metabolites therein. Additionally, results achieved from this assessment found that the type and duration of centrifugation and freeze–thaw episodes did not significantly affect results acquired.

As also noted in Ref. [1], one critical requirement is the employment of an oral activity abstention time threshold for the donation of WMS samples by study participants, and we find that an absolute minimum duration of 2–4 h, but preferably up to 8 h, is a pre-requisite for this. Otherwise, these samples will be contaminated, perhaps substantially so, by interfering levels of molecules arising from food intake (such as carbohydrates, lipids, lactate and citrate, amongst many others) [1], or other sources, for example methanol and other ^1^H NMR-detectable xenobiotics arising from the inhalation of cigarette smoke [9].

#### 2.1.2. Complications Arising from the ^1^H NMR Analysis of Metabolites with Exchangeable ^1^H Nuclei

It is also of much importance to note that selected presaturation pulse sequences such as the *noesy-presat* technique applied here, unfortunately can significantly suppress the intensities of exchangeable ^1^H nuclei resonances, e.g., that of the broad H_2_N-CO- proton signal of urea in spectra acquired, and therefore due consideration of this process is required prior to the direct and absolute quantification of metabolites in such spectra (although usually the acquisition of spectral profiles of a series of standard calibration solutions under the same experimental parameters and conditions is sufficient to overcome these problems). However, in the *noesy-presat* spectral profiles of human saliva, such as those acquired in Figure 1 here, only a very small fraction of the total number of resonances present arise from biomolecules with such exchangeable protons. However, use of the quite newly developed ROBUST-5 and, by extrapolation, WASTED pulse sequences (as reported in Ref. [1]) are reported to not significantly influence the intensities of such signals.

**Figure 2:**
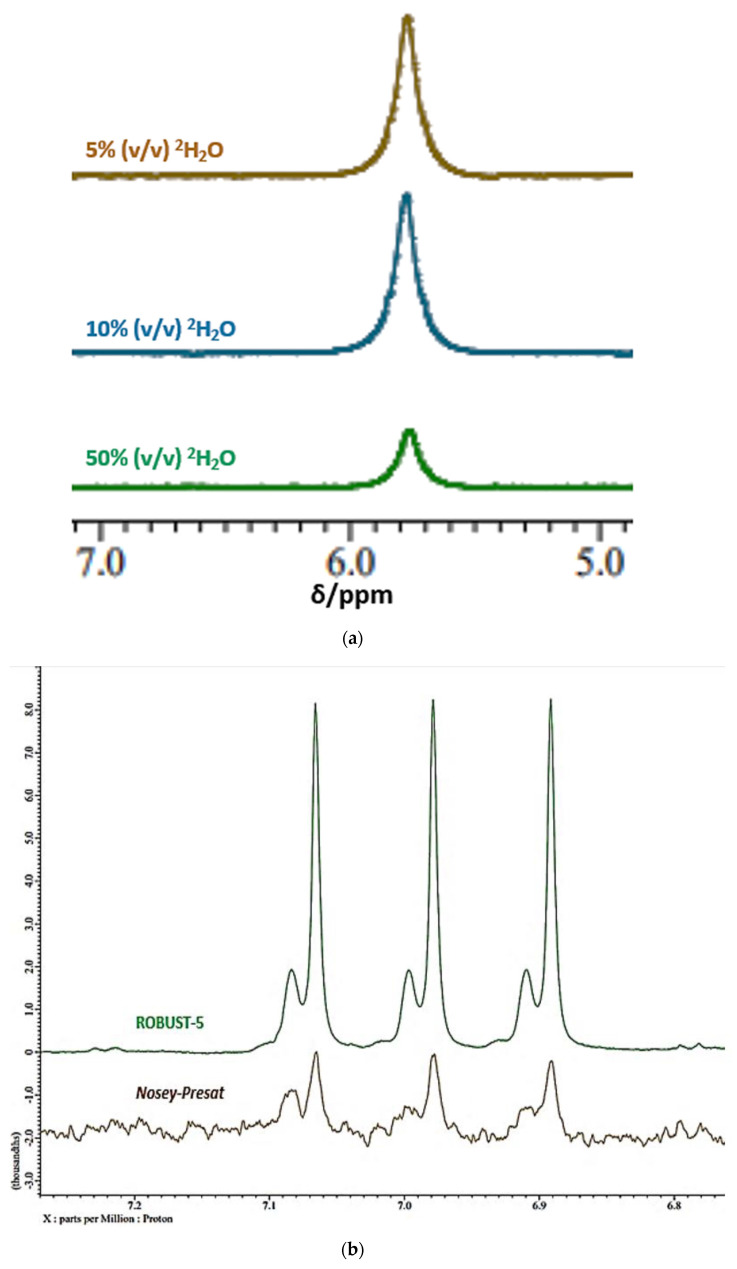
(**a**) **^1^H NMR spectra of urea at increasing added % (*v*/*v*) ^2^H_2_O solution contents.** Shown are the 4.90–7.10 ppm regions of spectra of 10.00 mmol./L urea acquired in solutions containing 5 (brown), 10 (blue) and 50% (*v*/*v*) ^2^H_2_O (green) using the Robust-5 pulse sequence. Samples also contained 10.00 mmol./L phosphate buffer (pH 7.00), and were equilibrated for a minimum duration of 1.0 h at ambient temperature (22 °C) prior to ^1^H NMR analysis. Spectra were obtained on a Jeol JNM-ECZ600R/S1 NMR spectrometer operating at a frequency of 600.17 MHz. Acquisition parameters were: sweep width 9 kHz; 16,384 data points; relaxation delay 1.0 s; 128 transients; and sweep width of 11,218 Hz. Broad urea resonance intensities were normalised to that of a TSP internal standard (final added concentration 125 µmol./L), with a chemical shift reference value set at δ = 0.00 ppm. Triplicate determinations were made for each ^2^H_2_O content, and typical spectra are shown. Estimated mean ± SEM ‘NMR-visible’ urea concentrations for these triplicate test solutions were 3.18 ± 0.020 mmol./L for 5% (*v*/*v*) ^2^H_2_O; 2.95 ± 0.021 mmol./L for 10% (*v*/*v*) ^2^H_2_O; and 1.28 ± 0.022 mmol./L for 50% (*v*/*v*) ^2^H_2_O. (**b**) **Direct ^1^H NMR analysis of ammonia as ammonium ion in human saliva.** Displayed are the 6.76–7.27 ppm regions of a WMSS sample showing signals emanating from the exchangeable protons of ammonium ion (NH_4_^+^) in aqueous acidic solution (final pH value 2.00) containing 10% (*v*/*v*) ^2^H_2_O, and using either the ROBUST-5 (green) or *nosey-presat* (brown) pulse sequences (pH values were adjusted with added HCl). Typical spectra are shown. The spectrometer utilised, and the spectral acquisition parameters involved were those given in (**a**) above. Resonance chemical shift values and intensities were normalised to that of a TSP internal standard (δ = 0.00 ppm). For this sample, the NH_4_^+^ ion concentration was estimated to be 7.60 mmol./L by application of a standard additions method.

Moreover, exchange of such ^1^H nuclei with ^2^H from ^2^H_2_O present in analyte solutions would be expected to give rise to a significant level of resonance attenuation irrespective of the pulse sequence employed, and Figure 2a shows the ^1^H NMR spectra of an aqueous solution of a standard solution of urea (10.00 mmol./L) at pH 7.00 acquired at increasing added aqueous solvent system contents of ^2^H_2_O (5.0, 10.0 and 50.0% (*v*/*v*)) using the Robust-5 pulse sequence. Clearly, increasing the ^2^H_2_O content of the solution medium gives rise to major reductions in the ‘NMR-visible’ concentration of urea, as expected. Interestingly, a plot of these replicated ‘NMR-visible’ urea concentrations versus % (*v*/*v*) ^2^H_2_O solution content was strongly negative and linear throughout this ^2^H_2_O content range (r = −0.99934, *p* < 10^−10^), although since the y (zero)-intercept for this plot was only 3.38 mmol./L and not the actual base starting concentration of 10.00 mmol./L, this relationship is not expected to hold within the 0.00–5.00% (*v*/*v*) ^2^H_2_O content span (further experiments to explore this are currently in progress). Confidence intervals (95%) for the regression coefficient (gradient) of this plot, with estimated ‘NMR-visible’ urea levels in mmol./L on the ordinate axis, were −0.043 to −0.041% (*v*/*v*) ^2^H_2_O content (data not shown).

A similar observation was made for the exchangeable ammonium ion (NH_4_^+^) protons when comparing standard pre-saturation with the ROBUST-5 pulse sequence (Figure 2b), the latter with a reportedly much more effective suppression of the main water signal feature, in addition to not attenuating exchangeable proton signals [10]. In contrast to the *noesy-presat* sequence, spectra acquired using this pulse sequence were reproducibly found to have a markedly greater intensity for the NH_4_^+^ ion signal in acidic solution (pH 2.00), which supports its use as a means to directly quantify this species, along with further metabolites containing exchangeable protons. With increasing added solution ^2^H_2_O (*v*/*v*) % contents, an additional splitting of NH_4_^+^ ion’s characteristic 1:1:1 intensity ratio ^1^H triplet resonance, which arises from ^1^H coupling to its central ^14^N nucleus, was observed. This additional splitting is ascribable to the sequential, ^2^H_2_O solvent content-dependent, generation of increasing levels of deuterium-substituted ammonium ions, and up to four NMR-visible species (NH_4_^+^, NH_3_D^+^, NH_2_D_2_^+^ and NHD_3_^+^) are produced in acidic aqueous media containing ≥50% (*v*/*v*) ^2^H_2_O (data not shown). The higher frequency minor signal shown in Figure 2b is predominantly ascribable to NH_3_D^+^, with successively lower amounts of higher deuterium-substituted species at this ^2^H_2_O level; of course, ND_4_^+^ is ^1^H NMR-invisible. To the best of our knowledge, this is the first reported determination of ammonia/ammonium ion in human saliva—these results will be reported in detail elsewhere.

Additional reviews focused on the collection, laboratory preparation, storage and ^1^H NMR spectral acquisition parameters and differential NMR techniques applied are available in [1,11,12,13].

In a quite recent fascinating report [14], Hauslauer et al. studied the influence of increasing ^2^H_2_O contents of NMR aqueous buffer solvents on the temperature exposition of human urine samples, and the effects of ^2^H_2_O equilibration time, on the ^1^H NMR spectra acquired on this biofluid. These researchers discovered a profound decrease in the urinary creatinine-CH_2_ signal, this amounting to up to 35% in intensity following a 24 h dwell period in a medium containing 25% (*v*/*v*) ^2^H_2_O. However, this intensity reduction was only 4% when the ^2^H_2_O content was only 2.5% (*v*/*v*). These observations were consistent with a ^1^H/^2^H exchange at the -CH_2_ position of this metabolite, and the application of inverse-gated (IG) ^13^C and DEPT-HSQC NMR analyses, along with MS experiments, were all fully consistent with this process. Therefore, this work had very important implications regarding the determination of creatinine in biofluids, including WMSSs, using aqueous solvent systems containing >2.5% (*v*/*v*) ^2^2H_2_O for NMR analysis purposes, most especially when samples prepared do not undergo equivalent dwell times prior to analysis. More critically, since this metabolite is very often used as a normalisation reference feature for NMR-based metabolomics experiments involving the ^1^H NMR analysis of urine, the bioanalytical significance of this observation is manifold. Indeed, without careful consideration and control of analytical matrix ^2^H_2_O contents, nor sample dwell times following preparation with this field frequency lock solvent, nor that of any further relevant conditions such as temperature, then its application as an ^1^H NMR normalization reference feature in future studies is clearly precluded. However, the authors of Ref. [14] devised a method to correct for such time- and condition-dependent urinary creatinine losses.

### 2.2. Mouth-Rinsed Water Washouts as an Alternative Diagnostic Medium to WMSS Samples

One potentially interesting proposal put forward by Maruyama et al. [15] featured the collection and analysis of mouth-rinsed water (MW), the rationale being that the collection of WMS under controlled protocols was defined by them as time-consuming and onerous, and hence apparently was unsuitable for use in large cohort investigations. According to the authors, MW may be easily collected in less time than that taken for WMS collection, and with ‘…less difference between subjects…’, whatever exactly that may indicate? Therefore, such samples are rendered more suitable for MV metabolomics analysis of patient cohorts. In order to investigate this, the researchers collected MW, along with both stimulated and unstimulated saliva samples, from n = 10 healthy control participants, and analysed each one using capillary electrophoresis time-of-flight mass spectrometry (CE-ToF-MS). Results acquired showed that qualitatively, MW samples contained the same metabolites as those detectable in stimulated and unstimulated salivary aspirations. Perhaps surprisingly, these authors found that the quantities of biomolecules monitored did not markedly differ between the three different sampling approaches, and that MW samples may act as suitable alternatives to WMS samplings for analysis of oral metabolome profiles.

In our view, application of this MW sample collection procedure should dilute or markedly dilute the concentrations of metabolites present in WMS, but would not necessarily modify the proportionate amounts each metabolite present, and hence, comparative data may be analysed in a MV context using the probabilistic quotient or constant sum normalisation approaches (PQN and CSN, respectively), and this would also allow for any differences in the water wash volume received by participants. This analysis strategy may therefore possibly be suitable for the collective study inclusion and/or comparison of MW and WMSS samples. Additionally, notable is the knowledge that even high-resolution ^1^H NMR analysis may not achieve sufficient sensitivity for the direct analysis of at least some biomolecular analytes in oral MW samples; however, the CE-ToF-MS technique employed in Ref. [11] may indeed have an adequate sensitivity limit of detection for a range of salivary metabolites in such diluted MW specimens.

### 2.3. Influence of Prior Oral Cavity Cleansing Episodes on the Metabolic Profile of Human Saliva

In 2021, Letieri et al. [16] conducted a cross-sectional clinical investigation coupled with a convenience sample in order to assess the influence of oral mucosa cleansing on the salivary metabolic profiles of infants in their pre-dental periods (mean age 3.8 months); these profiles were acquired through ^1^H NMR analysis on a 500 MHz spectrometer. Perhaps unsurprisingly, these researchers found that this oral cleansing approach, which involved the application of gauze moistened with filtered water, resulted in a decrease in glucose, lactose, further sugars, acetate, alanine and lactate, which may all arise from dietary sources anyway. According to the authors’ protocol, this would be expected, since pre-cleansing saliva samples were collected using an automatic pipette coupled with sterile plastic tips in the buccal floor area at time-points specified as only at least 1.0 h subsequent to the last feeding session. Indeed, this 1.0 h delay period is almost certainly insufficient to remove all dietary agents from these pre-cleansing saliva samples [1]. Moreover, post-cleansing samples were only collected at a time-point of 5 min following the completion of this process, and that may be insufficient to ensure the removal of all dietary-derived agents therefrom. A further limitation of this investigation is the small sample size (n = 11 participants); nine of these were exclusively breastfed, which may in itself affect the concentrations of any dietary-derived molecules in the samples collected, most especially if an insufficient fasting period is featured in the protocol prior to sample collection (as noted above).

### 2.4. Comparisons between the Metabolic Profiles of Whole, Parotid and Submandibular/Sublingual Saliva Specimens

Since biomolecules present in submandibular/sublingual saliva samples were not previously systematically analysed, one study conducted by Meleti et al. in 2020 [17] explored samples of whole, parotid, and submandibular/sublingual saliva collected from a total of 20 healthy human participants, who were free from any PDs and other dental conditions—samples were analysed by NMR analysis. These researchers successfully identified a series of metabolites which were differentially distributed amongst the three sub-types of saliva sample, specifically 54 in WMS, and 49 and 36 in parotid and submandibular/sublingual fluids, respectively. Application of PCA to the evaluation of these datasets indicated a clear-cut clustering for the profiles of WMS samples, which partially superimposed upon those representing both the parotid and submandibular/sublingual metabolite signatures. Exclusive biomolecules found for the three salivary sampling sub-types were 2-galactose, *iso*-caproate, hydroxy-3-methylvalerate, 3-methylglutarate, 3-phenylpropionate, 4-hydroxyphenylacetate and 4-hydroxyphenyllactate for WMS, arginine for parotid saliva, and caprylate and glycolate for submandibular/sublingual saliva. In conclusion, the authors further specified that all salivary biomolecules were segregated into four main classes: (1) proteinogenic and non-proteinogenic amino acids and amines, salivary taurine representing an example of the former (a β-amino acid containing a sulphonate function); (2) simple carbohydrates; (3) organic acids (as anions at neutral salivary pH values); and (4) bacterial-derived metabolites, combinations of which may provide useful biomarkers for oral and/or systemic diseases. However, conversely, not all the carbohydrates are simple (including those of the branched side-chains of acute-phase glycoproteins, and N-acetylated mono- and oligosaccharide species, etc.). Moreover, many organic acids, as anions at neutral or near-neutral pH values, are actually bacterial catabolites, although not exclusively so [1]. However, the recognition of a salivary gland-distinct, albeit limited, metabolite profile in healthy human populations as documented in this study may indeed be of some diagnostic value.

## 3. Diagnosis and Monitoring of Human Oral Diseases Using Metabolomics Approaches, NMR-Based or Otherwise

Short-chain organic acid anions such as lactate, propionate, succinate, pyruvate, acetate, *n*- and *iso*-butyrates, formate, 5-aminopentanoate and fumarate, amongst others, which are present in whole mouth saliva can serve as PD markers, and salivary levels of these biomolecules reflect the growth, dominance and metabolism of micro-organisms [18,19]. Indeed, it is conceivable that selected groups and patterns of these metabolites serve as chemotaxonomic markers of microbial infiltration, even though at least some of them are also involved in normal host metabolic routes. Indeed, the anaerobic pathogen *Porphyromonas gingivalis* produces high concentrations of *n*-butyrate [20]. N-acetylsugars may be generated from the bacterial enzymes hyaluronidase and/or neuraminidase [21], in addition to their biosynthetic route from glucose in vivo. Malodorous amines, including methyl- dimethyl- and trimethylamine monitored in human saliva, may serve as toxic agents formed from bacteria involved in the pathogenesis of PDs [22], although this remains to be further explored and verified. Furthermore, the molecular nature (most especially relative acidities), and concentrations of salivary organic acids, which act as tooth-eroding agents, may have valuable roles as markers of patients’ susceptibilities to dental caries [14,18]. Elevated oral cavity levels of volatile sulphur compounds (VSCs) found in patients with oral malodour also adversely contribute towards PDs [23].

### 3.1. Periodontal Diseases (Types 1–4)

#### 3.1.1. Gingivitis (Type 1)

Gingivitis (type 1) represents the first phase of gum disorder, and is the mildest form of PD. This condition involves an inflammation of the gums which features redness, swelling, and occasionally bleeding experienced during tooth-brushing or probing episodes. Pathologically, gums are assaulted and irritated by toxic agents generated by dental plaque and tartar bacteria that have assembled on both teeth and gums in view of poor oral hygiene control. Hence, gums involved become inflamed, and their attachment to teeth is loosened. This process also exposes enamel which was covered prior to this process. The sulcus, which is the distance between the gum and the tooth, widens, producing what is known as a periodontal pocket (pocket depths are typically 1–4 mm when monitored on periodontal probing). However, this primary stage of PD is treatment-receptive and hence rescuable, most notably since teeth involved remain robustly socket-planted without any associated damage to bone or connective tissue. Indeed, an improved level of oral hygiene, involving a more rigorous regimen of tooth-brushing sessions, and the use of oral rinse products, for example, can reverse this early stage of gum disorder. Tooth-scaling and root planning may also be employed as a treatment. Although it may exhibit little or no symptoms, gingivitis may readily progress onto the next stage of PD, early periodontitis.

Additionally, gingivitis can give rise to a condition which is commonly known as ‘trench mouth’, which is often caused by poor oral hygiene. However, other important causes are a poor dietary intake, general stress and sleep deprivation. ‘Trench mouth’ symptoms frequently appear swiftly, and these encompass fatigue, pain and fever, together with oral malodour. This condition gives rise to open sore development on the gums, and the demise of tooth-surrounding tissues; indeed, gums are susceptible to bleeding, especially during chewing episodes. Notably, the pain involved can prevent patients from eating or swallowing, and the inflammation involved may spread to local face and neck tissues.

#### 3.1.2. Early (Type 2) Periodontitis

Early (type 2) periodontitis involves the extension of inflammation and infection to tooth-supporting bone which, along with associated fibres, experience irreversible damage. Therefore, this is a more severe class of PD, and plaque bacteria penetrate further within the sulcus. Consequently, this environment allows the infiltration of anaerobic bacteria under the gumline, and gingival pockets are generated therein. Treatments for this early stage of periodontitis again usually involves tooth scaling and root planning, together with an improved degree of oral hygiene. Since bone loss at this stage is minimal, generally no further treatment is mandatory.

#### 3.1.3. Moderate (Type 3) Periodontitis

Moderate (type 3) periodontitis features a major infection of surrounding connective tissues and alveolar bone, which retain teeth in their place. Both bacterial toxins and human enzymes combating infection play important roles in this degradation process, and tooth mobility arises from bone loss. Key symptoms of this stage of the disease progression are gum recession and exposure of the root surface, along with root decay and tooth sensitivity; bleeding gums and persistent bad breath; and pocket depths of 6–7 mm and intermediate bone losses of 20–50%. Moreover, teeth loosening may occur, and periodontal abscesses may evolve. Since this represents a key critical phase of the condition, only surgical treatment may halt its progress; the damage caused is largely irreconcilable.

#### 3.1.4. Advanced (Type 4) Periodontitis

Advanced (type 4) periodontitis, the final stage of PD, is largely characterized by severe infection, loosening teeth and tooth loss. In most cases, these processes are so severe that the surgical extraction of teeth is a necessity, in order to prevent the spread of infection. Common further symptoms are spontaneous gum bleedings, continuous oral malodour, tooth sensitivity in view of root exposure, oral pus drainage from periodontal abscesses, pocket depths of >7 mm, and substantial bone losses (>50%). Therefore, there is a very poor prognosis with this final stage of PD, although an expansive programme of periodontal gum surgery may be employed to rescue affected teeth; this process may involve both soft and hard tissue grafting procedures.

Full clinical details regarding the diagnosis, classification and sequential severity of PDs can be found in Refs. [24,25].

#### 3.1.5. Salivary Metabolomics Investigations of PDs

In 2012, Aimetti et al. [19] explored the salivary ^1^H NMR profiles of generalised aggressive periodontitis (GAP) patients, and found that *n*-butyrate was significantly upregulated in this group over those of age-matched healthy controls, although this work also revealed that acetate, propionate, γ-aminobutyrate, succinate, trimethylamine, and the amino acids phenylalanine and valine, were also increased in salivary concentration in this group; downregulated pyruvate and acetamido-NH-CO-CH_3_ functions were also found. These results were supported by a study conducted by Rzeznik et al. [26], who also observed that ^1^H NMR-based metabolomics analysis of human saliva offered a valuable and robust probing system for the early diagnosis and subsequent prognostic follow-up of PDs. Indeed, this study found that regardless of the class of periodontitis (i.e., aggressive versus chronic), significant metabolic differences were found between saliva specimens collected from patients with these conditions and those of healthy controls. These abnormal patterns featured upregulated *n*-butyrate, along with downregulated lactate, threonine, γ-amino-butyrate and methanol, the *n*-butyrate representing a common and quite specific catabolite derived from the oral microbiome.

Chronic periodontitis is commonly associated with *Porphyromonas gingivalis*, and the invasion and proliferation of this species into the periodontium is strongly linked to metabolomic modifications in the oral cavity. *P. gingivalis* is a ‘red complex’ bacterium, and recently researchers have evolved an enzyme-linked immunosorbent assay (ELISA) test kit to identify *P. gingivalis* in human saliva [27], a development which may serve as a valuable chair-side diagnostic probe for its infiltration in PD and other oral conditions. The kit detects this bacterium at levels of 5 × 10^4^ to 5 × 10^5^ CFU/mL, and generates results rapidly (i.e., within 90 s), which is much faster than previously available polymerase chain reaction (PCR)-based systems; it has a reported specificity and sensitivity of 96 and 92%, respectively.

More recent metabolomics investigation of the diagnosis and progression of PDs are described below. Gawron et al. [28] conducted a quite unusual study involving the ^1^H NMR analysis of mouth washout and tongue swab specimens to explore the metabolic profile of the oral cavities in human participants with chronic PDs. In total, these samples were found to contain 23 and 17 metabolites, respectively, and as expected, many of these at least partially overlapped with those found in human saliva. However, when compared to results acquired on healthy control participants, bacterial colonisation of the oral cavities of PD patients was found to be associated with modifications in the concentrations of 8 biomolecules, specifically upregulated isopropanol, along with an ‘unknown’ unassigned resonance (with a triplet signal located at δ = 0.89 ppm), and downregulated glycerol for tongue swab specimens, whereas mouth washouts were found to have upregulated lactate levels, but also significantly decreased concentrations of tobacco smoking-relevant methanol, uncontrolled diabetes-generating acetone, and a further unknown metabolite (with a singlet resonance located at δ = 1.25 ppm). Although we were unable to review the ^1^H NMR spectra obtained on these samples, it remains a possibility that the unknown δ = 0.89 ppm triplet arises from dietary lipid-derived fatty acid (FA) chain terminal-CH_3_ resonance, and possibly another unassigned resonance centred at 1.29 ppm (multiplet) arises from bulk-acyl chain lipid-(-CH_2_-)_n_ signals; however, this pattern of resonances, together with the 1.56 ppm one (apparent quartet found in mouth washout spectra only), and/or the 3.71 ppm multiplet observed in tongue scraping spectra only, may be assignable to sodium dodecyl sulphate (SDS), or a related detergent. Notwithstanding, the unknown multiplet at δ = 3.02 ppm, also detectable in the mouth washout profiles, may be assignable to the 5-position-CH_2_ groups of the salivary bacterial catabolite 5-aminopentanoate, or the ε-position -CH_2_- group of its amino acid precursor lysine, or even the 4-position-CH_2_ protons of γ-aminobutyrate (all triplets), or a superimposition of two or more of these biomolecules, whereas that at δ = 3.71 ppm in tongue scrapings may also be ascribable to a saccharide species proton, the α-CH proton of an amino acid, or perhaps even the -CHOH proton of butane-2,3-diol.

One major concern of the above research report is that the mouth washout samples were collected through the rinsing of participants’ mouths with a fixed 0.50 mL volume of 0.90% (*w*/*v*) saline solution for a duration of 20–30 s, followed by collection of the sample. Although we appreciate that the same protocol was employed for every participant recruited, researchers would, of course, be completely unaware of the total volumetric contributions of saliva and other much less prevalent oral fluids (e.g., gingival crevicular fluid (GCF)) to the samples donated, and therefore without this information, which perhaps could have been obtained by employing any recommended pre-calibration and standardisation methods for the determination of salivary/other oral biofluid mass or volume contributions to the ‘washout’ samples provided, if indeed there are any, then any metabolomics data arising therefrom will not at all be comparable from sample to sample, irrespective of what sampling group they were in. To the best of our knowledge, no such standardised protocol has yet been fully established. Likewise, there was no consideration made for variable salivary flow-rates (SFRs)—in principle, the higher the SFR, the lower the actual salivary concentration of low-molecular-mass biomolecules, but conversely the larger the total salivary contribution towards the washout samples collected. However, fortunately the authors utilised probabilistic quotient normalisation (PQN) [29] as a sensible solution to this type of problem, which is encountered when there are unknown biofluid sample volumes used for analysis, or unknown contributions of actual biofluids towards the final sample donated or analysed, as in the washout samples collected in that study. The constant sum normalisation (CSN) approach also permits such variable or unknown volumetric sample size comparisons in a MV context. These normalisation methods are commonly employed in the preprocessing stage of metabolomics dataset analysis, and they allow for differing levels of ‘between-sample’ dilutions by scaling the intensities of spectral profile resonances to the same virtual overall total intensity (as in CSN), or alternatively to those of a pre-selected reference sample, or a representative ‘pooled’ (average) sample from one of the classification test groups, usually the healthy control or baseline one (as in PQN).

A very similar dataset normalisation consideration also applies to the tongue swab samples collected into phosphate-buffered saline (pH 7.2) in this study, where again PQN was employed before the performance of univariate and MV data analysis regimens. Nevertheless, one major drawback of this study was that no preservatives were added to the biomatrix specimens collected, which may result in the artefactual production and/or fermentative consumption of some biofluid constituents during episodes of transport, sample preparation and deep-freezer storage, or the sample collection process itself.

Lactate, which is generally recognised as a product of oral microbiome saccharide catabolism arising from poor oral hygiene [30], was found to represent a statistically important metabolite present in the mouth washout specimens. Indeed, in one investigation focused on generalised aggressive periodontitis [31], significantly upregulated levels of this organic acid anion were found in saliva over those of a control group, whereas formate concentrations were lower, and these observations were linked to *P. gingivalis*—positive sites.

In 2018, Chen et al. [32] also investigated generalised aggressive periodontitis (GAgP) in human patients, but this study explored the metabolic profiles of blood serum and GCF rather than saliva, since at that point in time the pathogenesis of this condition remained unclassified. Hence, this study set out to identify any differential metabolic profiles in these biofluids between such patients (n = 20 participants) and those of an equivalent number of healthy controls in a GC/MS-based untargeted metabolomics approach. They discovered that GAgP patients had significantly upregulated serum concentrations of urea and allo-inositol, and downregulated concentrations of glutathione, 2,5-dihydroxybenzaldehyde, adipate and 2-deoxyguanosine levels when compared to the profiles of healthy controls. However, GCF concentrations were shown to have significantly increased noradrenaline, uridine, α-tocopherol, dehydroascorbate, xanthine, galactose, glucose-1-phosphate and ribulose-5-phosphate levels, and significantly decreased thymidine, glutathione and ribose-5-phosphate concentrations in the GAgP group; the enhanced dehydroascorbate and reduced glutathione levels indicate a redox imbalance, which may be associated with oxidative stress in this disease process. These data suggested that such metabolomics studies offer much potential for the detection of GAgP, and in providing valuable information regarding possible mechanisms available for its induction and development.

In view of the promising potential offered by metabolomics analysis featuring GCF as an analytical medium, a related review [33] concluded that the simultaneous monitoring of both host and microbiome contributions and interactions in relation to the onset of PDs will serve to broaden our scope for the detection, measurement, and application of relevant, possibly inflammatory biomarkers, which may indeed arise from host inflammatory responses to bacterial challenges. Validation of such biomarkers will, of course, require proof of their reliability through the observation of reversals of disease-induced up- or downregulations in their biofluid concentrations, which result from the treatment of these conditions with already established therapeutic agents (i.e., those which are known to exert favourable effects against PDs).

This year, Balma et al. [34] conducted a systematic review of publications available on the metabolomics-linked diagnosis of PDs, with special reference to the quality assessment of methods available. These studies were identified from the *MEDLINE* (*PubMed*), *Embase* and *Scopus* databases, and their analytical protocols, significant biomarkers and results arising from MV metabolomics analysis techniques applied were systematically reviewed. A total of 12 investigations satisfied the selection criteria for entry into the study, and these had participant sample sizes which varied from 19–130. The authors then conducted pathway analysis using *MetaboAnalyst 5.0* online software (University of Alberta Cells 2021, 10, 572 6 of 53 and National Research Council, National Institute for Nanotechnology (NINT), Edmonton, AB, Canada) www.metaboanalyst.ca (accessed 5 July 2022); moreover, quality assessments of studies were performed using a revised version of the QUADOMICS tool. This review found that the branched-chain amino acids (BCAAs) isoleucine and valine, the aromatic amino acids phenylalanine and tyrosine, and the bacterial catabolite *n*-butyrate, were significantly upregulated in patients with PD in most investigations performed, whereas pyruvate, lactate and N-acetyl function resonances represented the most highly significantly expressed ‘markers’ in non-PD controls. Metabolic pathway analysis found that those which were most significantly perturbed were involved in inflammation and immune activation as we might expect, although dysfunctions in oxidative stress-and bacterial energetic metabolism-implicated pathways were also found. Overall, the authors concluded that PD was characterised by a ‘specific’ metabolic pattern in saliva (coefficients of determination ranged from 0.52–0.99). However, the authors also correctly suggested that it is essential that such biomarker signatures and metabolic pathways indicated from these studies would have to be correctly validated, and the only way to realistically do this is by seeking reversals of such biomolecular patterns, reflecting favourable clinical responses arising from the applications of already established therapeutic agents or regimens which are known to be effective for tackling PDs. Currently, however, this may only be possible for type 1 PD (gingivitis). Once fully validated, such candidate biomarkers may also permit the diagnosis of PDs, and the identification of previously undiscovered therapeutic targets for treatment.

Another recent report [35] featured a characterisation of the microbial population and metabolic profiles in GAgP. For this purpose, GC/MS-based metabolomics analysis was performed in conjunction with high-throughput 16S ribosomal RNA (rRNA) gene sequencing; overall, 146 sub-gingival plaque samples, along with 50 GCF specimens were collected from 24 GAgP patients and 10 PD-healthy participants. Intriguingly, some very profound differences were found between the sub-gingival microbiome and GCF metabolite patterns between these two groups of participants; however, no differences were observed between samples as a function of differential probing depths. Indeed, both metabolomics and enrichment analyses revealed that GAgP significantly modulated the levels of metabolites linked to amino acid biosynthesis, including that of alanine, and all BCAAs; galactose metabolism featuring *myo*-inositol, galactose, glucose, and hexitol; and pyrimidine metabolism, for example pathways involving uracil, uridine, β-alanine, and thymine. Interestingly, genera which displayed significant differences between the GAgP and healthy control categories were significantly correlated with further metabolites, although glucose and oxoproline were found to have the highest correlations with microorganisms. Therefore, these results showed favourably distinct microbial colonies and metabolic profiles between these clinical groups, and significant correlations observed between microbial taxa and salivary biomolecule concentrations indicated plausible mechanisms for periodontitis. The authors also concluded that their investigations served to offer valuable strategies for the detection of PD and the management of periodontitis.

Finally, the volatile organic compounds (VOCs) analysis of exhaled human breath has been markedly promoted by the adoption of major advances in the bioanalytical and instrumental research areas, and this strategy now stands at the frontline in clinical diagnostics and prognostic disease monitoring. Indeed, human VOCs emanating from human saliva, and/or present in exhaled breath such as acetoacetate-derived acetone from poorly or uncontrolled diabetic conditions, can, in principle provide useful information concerning disturbances in metabolic pathways. This area is not further considered here, but an invaluable review of the potential of salivary VOCs as monitoring probes for oral diseases such as dental caries, PDs including gingivitis, and oral cancer, is provided by Pereira et al. [36]. Moreover, this review also covers the current status of what is described as ‘salivary volatomics’, including relevant extraction protocols, current challenges and future outlooks for such strategies.

Further information focused on the use of ^1^H NMR-based metabolomics techniques for monitoring the pathophysiological status of PDs, albeit in conjunction with types 1 and 2 diabetes, is considered below in Section 4.2.

Bostanci et al. [37] explored and reviewed the development of modern day high-throughput technologies for the comprehensive and simultaneous determination of many biomolecules simultaneously in ’state-of-the-art’ ‘omics technology research areas. Indeed, such developments have facilitated the collection of ’big datasets’ to characterize the inputs of many different sources of variation, be they microbial, host metabolic pathway, or participant demographic, etc. The aim of this study was to concentrate on both metabolomics and metaproteomics strategies to facilitate the performance of pivotal studies on a range of samples collectable from the oral environment. Therefore, a composite of both proteomics and metabolomics techniques as described in this study was found to complement determinations of the functions and basal regulatory mechanisms involved in different oral microbial communities, for example their interactions within oral biofilm milieux. The authors discussed these issues with respect to both scientific and clinical standpoints, and communities arising from or indicative of PDs, dental caries, endodontic lesions and healthy control environments were considered. They also presented possible challenges, future prospects and examples of best practice.

To date, some research investigations have attempted to establish a positive association between periodontal and pulmonary diseases. However, since this association continues to require clarification, Wu et al. [38] conducted a full meta-analysis in order to explore associations between periodontal health and some common pulmonary disorders, specifically chronic obstructive pulmonary disease (COPD), asthma, and pneumonia. To achieve this aim, the researchers involved searched no fewer than seven sites for all studies featuring relationships between periodontal and pulmonary diseases. Weighted mean difference (WMD)/odds ratio (OR) parameters with associated 95% confidence intervals (CIs) were utilised to evaluate the power of these co-dependencies. This study found that of the 37 investigations retained for this meta-analysis, a pooled model approach revealed significant relationships between periodontal and pulmonary disorders (adjusted OR 1.93, with pooled adjusted OR values of 1.64, 2.21 and 3.03 for COPD, pneumonia and asthma, respectively). Therefore, the pooled analysis conducted found that pulmonary disease patients experienced lower levels of periodontal health; indeed, a high proportion of PD index scores for these patients were higher. Future clinical trials for evaluating the causality and pathology of relationships between these diseases were suggested to be required.

In a further study, Na et al. [39] investigated the oral microbiome and metabolome in order to provide data on associations of the former with metabolites present in oral fluid samples collected from sub-groups of PD patients. For this purpose, they recruited a large number of participants (n = 112), and collected buccal and supragingival samples for microbiome analysis, and saliva for metabolomic analysis. Bioinformatics strategies were employed for a combined integrated analysis of these datasets. Results acquired provided evidence for the involvement of 28 biomolecules for the distinction of PD disease patients from those of the healthy control group. The PD group was then additionally sub-clustered into two sub-groups according to their metabolic patterns (PT_G1 and PT_G2), and oral microbiome analysis found significant discriminatory bacterial species within these two classifications of microbiota. Indeed, the PT_G2 class contained higher levels of short-chain FAs with a greater abundance of pathogenic bacteria. Hence, this work demonstrated powerful relationships between the oral metabolome and microbiome. Moreover, the employment of multi-omics strategies in this manner may provide valuable diagnostic biomarkers for PDs, which, in turn, may lead to the prediction of improved treatment options for these conditions.

### 3.2. Dental Caries

#### 3.2.1. Analysis of Primary Root Carious Lesion Biopsies

Although most NMR-based metabolomics investigations have focused on the multicomponent analysis of human saliva, and fewer still with alternative oral fluids such as GCF, some limited studies have also involved the analysis of suitable extracts derived from solid clinical biopsies such as plaque, carious dentin and extrinsic tooth discolouration matrices. Clearly, such investigations are more relevant and valuable, since they are directly targeted at and sample from the site of pathological damage. Indeed, saliva may only present a diluted or highly diluted ‘picture’ of such potentially uncharacteristic, pathologically modified metabolic patterns. Moreover, the analysis of GCF offers many advantages over saliva since it supplies localised site-specific information on the inflammatory status of tooth gingival margins in, for example. PDs. However, in view of the limited, often sub-microlitre quantities of GCF collectable from the oral environment, most previous metabolomics studies of this fluid have employed more sensitive LC/MS or GC/MS analytical platforms which are able to cope with such volumes, unlike solution-state NMR analysis. At the very first IADR symposium focussed on the applications of high-resolution NMR analysis in the oral sciences in New Orleans, LA, USA in 2007, the applications of this technique to the metabolic profiling and fingerprinting of carious dentin, plaque and extrinsic tooth discoloration biopsies, and their prospective long-term research potentials, were presented [40].

However, prior to this, in 1999 Silwood et al. [22] reported on the high-field ^1^H NMR analysis of post-neutralized HClO_4_ extracts of active primary root carious lesions, particularly the organic acid status of these biopsies since, together with diminished pH values, the molecular nature and concentrations of these agents represent key demineralisation indices in the chemopathogenesis of dental caries. Results acquired demonstrated that the mean ± SEM percentage biomolecule content values of these lesions were acetate 51 ± 2%; formate 37 ± 2%; lactate 5 ± 1%; propionate 3 ± 0.8%; pyruvate 2.4 ± 0.3%; *n*-butyrate 1.2 ± 0.2%; succinate 0.1 ± 0.1%; and *iso*-butyrate, *n*- and *iso*-valerates, and *n*- and *iso*-caproates (total) <0.2%. Further ^1^H NMR-detectable features included alanine, choline, glycine, glycolate, methanol, phosphorylcholine and trimethylamine oxide, along with various fermentable saccharides. From these results, it was concluded that in addition to lactic acid, both formic and pyruvic acids may significantly contribute to the lowered pH values of active caries lesions, since they have quite high acid dissociation constants (*K*a = 1.77 × 10^−4^ and 3.20 × 10^−3^ mol./L, respectively; that for lactate is only 1.40 × 10^−4^ mol./L). Hence, formic and pyruvic acids may represent key demineralizing agents featured in the induction, development and adverse progression of dental caries.

As expected, the infiltration, levels, preponderance and oral environment localisations of both aerobic and anaerobic bacteria exerts a substantial influence and the distribution and potential chemopathogenic actions of organic ions generated therefrom [3,41,42]. Indeed, along with lactic acid/lactate, the inclusion of acetic acid/acetate as a key contributory species toward carious lesion development and progression was not unexpected in view of its established cariogenic potential [43]. The high levels of this agent found in the lesions analysed suggested only a limited availability of exogenous saccharides [44], together with a major permeation of collagen-deteriorating and amino acid-fermentative bacteria [19], e.g., *Arachnia* and *Propionibacterium*, which are often detectable within carious dentin [45]. These species contain enzymes with the abilities to degrade gelatin, a process which produces acetate, together with propionate and *n*-butyrate, organic acid anions all found in the carious dentin samples analysed. Saliva was also collected from these patients for ^1^H NMR analysis in this very early study, but the results acquired were not compared to those of an orally and dentally healthy control cohort of participants.

#### 3.2.2. Analysis of Human Saliva for the Diagnosis and Prognostic Monitoring of Dental Caries

In paediatric dentistry, salivary metabolomics investigations have provided valuable information regarding potential biomarkers for dental caries [46]. Those identified included upregulated amino acids alanine, aspartate, glutamine, glycine, isoleucine, leucine, taurine, tyrosine and proline. Downregulated *n*-butyrate was also observed in these paediatric patients; however, it was recommended that this observation should be treated with some caution, since covariables associated with the investigation, such as gender and dentition stage, may also contribute towards its salivary concentration [46]. Interestingly, this cross-sectional investigation also compared the impacts of stimulatory and non-stimulatory sample collection conditions, and collection via passive drool with the employment of an absorbing device. Both univariate and MV methods of data analysis were used to determine the statistical significance of differences observed for these comparisons.

A further study revealed that increased salivary *n*-butyrate, lactate, FAs and acetate levels in children with caries when they were compared to those of healthy controls [47]; reduced levels of phenylalanine, propionate, and saccharide species were also found. In a follow-up study, these authors explored the use of salivary NMR-based metabolomics techniques in order to determine the therapeutic effectiveness of a dental caries treatment regimen prior and subsequent to application, and some encouraging results were obtained [48]. Very recently, Da Silva et al. [49] investigated the oral health status of infants aged 0–28 months and that of their mothers during the breastfeeding term, and in this study, results acquired through the multicomponent analysis of saliva using NMR-linked metabolomics were successfully correlated with those arising from on-site clinical examinations. Data obtained revealed highly distinctive differences between infants with and without teeth (aged <6 months for the latter group) [49]. Indeed, the former group showed upregulations in the levels of a large number of salivary metabolites. Fortuitously, these data also provided evidence for the successful applications of such NMR-based metabolomics techniques for evaluating oral disease risk in paediatric dental patients.

For the study reported in Ref. [49], a passive drool protocol was recommended for the collection of WMS specimens, rather than the employment of swab applicators for saliva collection, since such applicators severely altered the NMR-linked metabolomic data acquired. We too, have noted this problem, and hence we also recommend the passive drool method. The avoidance of swab applicators or other materials or agents utilised as salivary stimulants has been previously stressed by Granger et al. [50], so that interferences arising from the chemical agents involved, or those embedded in the applicator devices, may be completely circumvented.

Hence, swab collection processes have the ability to give rise to highly significant changes in sample composition, this being highly consistent with our group’s observations. Indeed, we have found that the use of such collection devices releases quite large amounts of exogenous agents into the salivary medium, these typically including acetate, formate and glycerol contaminants, along with many further exogenous agents. Notably, ^1^H NMR profiles of aqueous extracts of these swab materials, i.e., those derived from their equilibration with phosphate-buffered HPLC-grade water were found to be very ‘busy’ indeed, and these many contaminant signals detectable precluded the measurement of most, if not all, salivary metabolites using this technique.

Furthermore, the influences of gender and dentition stage as potential explanatory variables were found not to be statistically significant, although that of dental caries was found to exert effects on a total of 21 output metabolite variables. These included selected amino acids and monosaccharides, observations providing some evidence for the involvement of protein hydrolysis and deglycosylation phenomena in paediatric caries. Hence, from this study, it appeared that unstimulated passive drool samples conveyed a significant caries metabolite pattern in children.

Notwithstanding, Pereira et al. [46] did not find any significant differences between unstimulated and stimulated saliva samples, the latter via prior mastication on paraffin wax pellets for a period of 3–5 min, with passive drool being indicated as a preferential method. However, the ^1^H NMR profiles of one alternative applicator used by the authors to enhance WMS sample collection after soaking in a phosphate-buffered aqueous solution free of any other agents contained no less than 60 or so resonances throughout the 0.8–7.8 ppm spectral region.

An up-to-date review of the current status of salivary NMR-based metabolomics investigations for the investigation and prognostic monitoring of oral and dental health, with special reference to early diagnosis, can be found in Ref. [11].

In 2021, Rosa et al. [51] conducted an experimental in vivo study to assess longitudinal modifications in the bacterial microbiome and metabolome potentially corresponding to the progression and apprehension of enamel caries lesion. In order to achieve this, natural caries activities in n = 3 consenting caries-free human participants before the performance of four premolar extractions for orthodontic purposes were monitored. This study involved the placement of a modified orthodontic band on smooth surfaces, and a mesh on those which were occluded. The caries-promoting protocol was applied for durations of 4 and 6 weeks, and following this a caries lesion arrest episode was conducted through a two-week toothbrushing episode (lesions were clinically confirmed, with quantification performed by micro-CT enamel density determinations). A 16S rRNA gene Illumina sequencing system was employed to evaluate the microbial composition of biofilms, and ^1^H NMR analysis was employed for metabolomics investigations. This work provided evidence that the progression of caries lesions and biofilm maturation were associated with enhancements in the numbers of Gram-negative anaerobes, including *Veillonella* and *Prevotella* species. Although the progression of caries lesions was correlated with the infiltration of *Streptococcus*, a more equivalent spread of *Streptococcus*, *Bifidobacterium*, *Atopobium*, *Prevotella*, *Veillonella* and *Saccharibacteria* (TM7) were associated with caries arrest. Notably, the ^1^H NMR-detectable metabolites acetate, lactate, pyruvate, alanine and valine, together with selected sugars, were found to be more abundant in mature biofilms when compared to those found in the early (newly formed) type. From these experiments, the authors concluded that their longitudinal bacterial microbiome and metabolomics results acquired served to provide some valuable mechanistic information regarding the involvement of biofilms in the progression and arrest of dental caries. The molecular biomarkers identified here are promoted as useful candidates for future validation testing studies.

## 4. Extra-Oral (Systemic) Diseases

### 4.1. Bioanalytical Considerations for Extra-Oral Diseases

Blood constituent biomolecules may enter salivary tissues through transcellular or paracellular pathways, and these involve passive/active transport and extracellular ultrafiltration mechanisms, respectively [52]. Indeed, salivary glands are very permeable and are surrounded by blood capillaries, which permits the release and exchange of blood-borne molecules into acinus cells, which form saliva [53]. Therefore, blood biomolecules, including both low-molecular-mass metabolites and proteins, and any xenobiotics such as drugs present, may indeed have the ability to exert a significant effect on the molecular composition of oral fluids. Hence, since it appears that biomarkers of disease activity in the systemic circulation are absorbed by the salivary glands, it is therefore feasible that saliva may be employed as a biofluid medium for the detection and perhaps prognostic monitoring of extra-oral diseases, as well as oral-based conditions. Hence, saliva has the capacity to transfer valuable information regarding the extra-oral health status of individuals, at least in principle, and with due consideration given to their final salivary concentrations, and the sensitivity of the bioanalytical techniques employed.

### 4.2. Types 1 and 2 Diabetes

Type 1 diabetes (T1D) has also been assessed using NMR-based salivary metabolomics [54]. Indeed, paediatric patients with uncontrolled type 1 diabetics, and those with hyperglycaemia (blood glucose concentration >11.1 mmol./L) were found to have higher concentrations of salivary lactate, acetate and sucrose, and lower levels of succinate when compared to healthy controls. From this study, the researchers involved concluded that the disease-induced perturbations to salivary metabolic profiles observed arises from imbalances in energy metabolism and insulin release. However, some questions arise from this study. Firstly, the detection of salivary sucrose may simply reflect an insufficient fasting/oral abstention period for participants, as noted in [1]. Secondly, Q^2^ (cross-validation goodness-of-predictive-fit) values obtained from their PLS-DA model applied were quoted as either 0.02 or 0.06, so how can there possibly be an effective distinction arising between the two groups evaluated? Thirdly, in hyperglycaemic T1D patients, we may expect to find that ketone bodies, particularly acetone, would be detectable in human saliva, especially since it is well known that the smell of this volatile lipid metabolite, which arises from the spontaneous decomposition of acetoacetate, is often detectable organoleptically by humans [55]. A further comment is that these researchers also attempted to determine disturbances to human patient metabolic pathways, but how exactly is this possible when the salivary microbiome, both saccharolytic and proteolytic routes, are responsible for a significantly large proportion of the quantities of salivary metabolites detectable, most notably when some of these anyway appear to be largely attributable to only one of these sources, e.g., propionate, *n*-butyrate and *n*-caproate to bacterial sources, and citrate and urea to an endogenous human one? However, from the PCA loadings vector analysis conducted in that study, and the work conducted by Gardner et al. [56], it is clear that some of these metabolites, particularly selected amino acids, may arise from both microbial (proteolytic bacteria for amino acids) as well as endogenous sources. Software available for such pathway analysis requires prior specification of the biological species for metabolites and pathways considered, e.g., *Homo sapiens* or *Rattus norvegicus*, etc., along with selected, albeit limited, microbial sources, and hence its application is clearly limited when applied to bi- or even multi-sourced salivary metabolites and their concentrations.

A further study involved an investigation of salivary gland dysfunction biomarkers in type 2 diabetes (T2D) patients, specifically to explore associations between the extent of metabolic control in participants with this condition and both qualitative and quantitative indices of disease-induced salivary modifications, which serve as indicators of salivary gland dysfunction [57]. For this purpose, a very satisfactory and realistic sample size of n = 74 T2D patients provided a single sample of unstimulated saliva, and salivary total protein levels and flow-rate, and xerostomia were monitored in each participant involved. Xerostomia was monitored using the validated Fox test which comprises assessments of patients’ subjective complaints, e.g., oral dryness, difficulty swallowing, burning mouth sensations, sensitivity to acidic/spicy foods, problems speaking without additional liquid supplements, and generalised taste changes.

In Ref. [57], the researchers involved found a positive association between the level of T2D metabolic self-control measured via glycated haemoglobin (HbA1c), and the total protein concentration in saliva specimens determined via a Coomassie blue staining method (Spearman’s correlation coefficient (ρ) 0.33, *p* = 0.004). However, an inverse correlation was found between HbA1c levels and pH (ρ −0.23, *p* = 0.05). From this investigation, the authors concluded that total salivary protein level, and perhaps also salivary pH, may be valuable for the evaluation of glandular dysfunction and damage in T2D patients. However, these correlations were clearly not very strong.

Since T2D and PDs appear to be bidirectionally linked, another study [58] was focused on the metabolomics recognition of molecular patterns for periodontitis that could permit the identification and establishment of biomarker features which may facilitate the diagnosis and prognostic monitoring of both conditions simultaneously. Almost consistent with our protocols, participants were requested to refrain from eating or drinking (excluding water) from 11 pm the previous night, and to ensure that tooth-brushing was conducted the night prior to but not on the morning of saliva sample collection (minimum volume 0.5 mL). Blood (minimum volume 10 mL) was collected following saliva sample donation. Collection of WMS samples involved sterile polypropylene tubes; samples were immediately frozen thereafter, and then stored at −80 °C until ready for transport to the laboratory for analysis. Metabolomics profiling was performed subsequent to sample metabolite extraction—extracts arising therefrom were analysed by both GC/MS and LC/MS techniques. All chromatographic separations were superseded by full-scan mass spectroscopic analysis to monitor and quantitate all ions detectable. This cross-sectional investigation involved the multi-feature analysis of as many as 161 coupled saliva and plasma samples collected from participants. Diabetic and non-diabetic participant cohorts were sub-divided into three groups, namely periodontally healthy, gingivitis and periodontitis. Results acquired revealed that differences between the salivary metabolic profiles of healthy, gingivitis and periodontitis recruits within the non-diabetic group included upregulated biomarkers of purine metabolism, cellular energetic stress and GSH metabolism (elevated levels of oxidised glutathione (GSSG) and cysteine-glutathione disulphide), the latter also indicating disturbances in redox status and oxidative stress. Further modifications detectable included additional upregulated oxidative stress biomarkers such as the purine deterioration metabolites inosine and guanosine, increased salivary amino acids indicting protein degradation, perhaps by proteolytic bacteria, and enhanced docosapentaenoate, linoleate and arachidonate FA patterns.

However, differences observed between the salivary biomolecular profiles between the T2D and non-diabetic groups also revealed alterations in carbohydrate, lipid and oxidative stress metabolite patterns in the diabetic samples. Intriguingly, the characteristic metabolic pattern associated with T2D was found to replicate that of PD development in non-diabetic subjects. Moreover, further distinctive metabolic signatures were linked to PDs in T2D patients.

Experiments performed on plasma samples, however, were limited to those featuring the measurement of sensitive glycaemic control markers, i.e., 1,5-anhydroglucitol (1.5-AG), 2-hydroxybutyrate and glucose, along with a comparison of these results with those obtained on saliva in both T2D and non-diabetic participants. As expected, T2D patients had very highly upregulated plasma glucose and 2-hydroxybutyrate levels, and markedly downregulated 1.5-AG concentrations. Saliva results were consistent with these observations, but for 1,5-AG and glucose, these differences were much less statistically significant; conversely, differences between 2-hydroxybutyrate levels were found to be more significant for saliva.

Overall, the authors of this extensive study concluded that selected metabolic signatures effectively distinguished between the PD groups explored, and the results obtained may be of some value for the evolution of diagnostic and therapeutic approaches customised to diabetic patients.

However, one major limitation of such studies is a clinical difficulty arising from the establishment of an acceptable abstention period in which participants are required to abstain from all oral activities before WMS or other oral fluids are collected for analysis. Indeed, diabetic patients require careful monitoring of carbohydrate intake on a regular basis, and therefore the instigation of a sample collection protocol which involves a fasting period of ≥8 h is not clinically feasible, nor acceptable. Hence, it may be necessary to limit this abstention duration to only one or perhaps two hours in such cases. Similar arguments may indeed apply to a series of other disease-afflicted participant populations, notably for ethically high-risk studies involving sensitive subjects who are generally viewed to be unable to provide written or verbal informed consent for themselves, such as those with neurodegenerative diseases, or paediatric populations aged <18 years.

In principle, the effects of diet-induced changes to the salivary metabolome could be avoided by the removal of known dietarily-sourced (e.g., glucose, sucrose or citrate, etc.) or dietarily-induced (e.g., lactate, etc.) biomolecules from the salivary profiles, or alternatively via the isolation and subsequent disregard of an orthogonal PC containing correlated metabolites all arising or biosynthesised from dietary sources. However, it is recommended that such studies should be ideally conducted through the feeding of a fixed standard diet to all study participants for an adequate time period (say, at least several days); these participants should, of course, also include the age-and gender-matched healthy control group(s). Once the effects of such diets on the metabolic profiles of human saliva samples are fully established, metabolomics researchers may then be in a promising position to seek disease-specific biomolecular contrasts between healthy and diseased groups of participants, and the recognition, further testing and validation of suitable biomarkers for the condition(s) investigated.

### 4.3. Cardiovascular Diseases

Although, to the best of our knowledge, salivary NMR analysis has not yet been applied to the possible diagnosis of cardiovascular diseases (CVDs), many non-NMR-detectable biomarkers, predominantly protein species, have been monitored, and published data reporting these bioanalytical strategies, and their application in acute-phase settings, was very recently reviewed by Bahbah et al. [59]. This review found that selective salivary biomarkers such as C-reactive protein, troponin-1, creatinine kinase myocardial band, and myoglobin all showed some biomarker potential, and had discriminatory diagnostic values that were similar to those of blood serum. Further potential biomarkers tested (potential oxidative stress biomarkers, myeloperoxidase and brain natriuretic peptide), however, provided inconclusive results. From these experiments, the authors concluded that although selected salivary CVD biomarkers offer some valuable clinical monitoring potential, this diagnostic approach remains in the early phases of development, and therefore additional investigations are required to validate the results acquired, and to ascertain threshold cut-off values for such biomarkers. Comparisons of results with those resulting from the evaluation and testing of already established biomarkers are also required. Intriguingly, Kosaka et al. [60] monitored the concentrations of salivary inflammatory cytokines (IL-1β, IL-6, TNF-α and prostaglandin E2), and found that these were significantly upregulated in atherosclerosis-based conditions and PDs, and therefore they may have use as biomarkers for the diagnosis of both these conditions.

Despite the unavailability of reports on the potential use of low-molecular-mass NMR-detectable salivary biomarkers for the diagnosis and prognostic monitoring of CVDs, this remains a significant and clinically important area for exploitation. Interestingly, salivary concentrations of α-2-HS-glycoprotein have been found to be significantly reduced in CVD patients, and this indicates that the highly disease-susceptible and variable signal intensity ^1^H NMR spectral region, which contains the acetamido function (-NHCOCH_3_) resonances of N-acetylsugars present in the molecularly mobile side-chains of salivary glycoproteins (δ = 2.03–2.09 ppm [1,2]), and ‘free’ N-acetylsugars and N-acetylamino acids, may offer some potentially profitable diagnostic information for CVDs, along with associated peptidome data [61].

### 4.4. Sjörgen’s Syndrome

Sjögren’s syndrome (SS) represents a chronic systemic autoimmune disease with the distinctive features of keratoconjunctivitis sicca and xerostomia. Further progression of SS leads to a diminished SFR accompanied by a corresponding dehydration of patients afflicted. Furthermore, there are reports of modifications in salivary constituents. However, there are also significant disease-induced modifications to the proteome and transcriptome in patients with SS, but to date the great majority of biomarker detection and monitoring studies are limited to those not involving the NMR-based metabolomics technique. Concentrations of the cytokines IL-4, IL-5, along with cytokine clusters, have been suggested to be valuable for the diagnosis of SS [62], although the application of such widely used inflammatory markers for a specific disease process remains questionable. A further investigation identified a total of 19 genes that were closely associated with the pathology of this condition, and which were related to phenomena including antigen presentation, lymphocyte osmosis and the induction of interferons [63]. Another study has found a series of biomarkers which are all upregulated in primary SS patients [64], and this encompassed the protein markers cathepsin D, β2-microglobulin and α-enolase, the mRNA markers guanylate binding protein 2, low-affinity IIIb receptor for IgG’s Fc fragment, and myeloid cell nuclear differentiation antigen.

Notwithstanding, to date there are only a very limited number of reports documenting the multicomponent analysis of salivary small molecules in SS, and as noted above, even less so reported on NMR-based metabolomics. Mikkonen et al. [65] reported on salivary levels of ^1^H NMR-detectable biomolecules in patients with primary Sjorgen’s syndrome, and found that a grand total of 7 out of 24 of these were significantly upregulated in this condition, and none were reportedly significantly downregulated. These upregulated metabolites included choline and taurine (*p* = 0.001 and 0.006, respectively), although their concentrations were found to be negatively correlated with salivary flow-rate, and this observation is, of course, consistent with hyposalivation and the dehydration status of patients with SS. However, it is not entirely clear why no inverse associations between the levels of other salivary metabolites and SFR were detected. Further, non-SFR-dependent upregulations involved alanine and glycine, and phenylalanine, proline and *n*-butyrate were also observed, the latter three being only marginally higher in concentration than those of healthy controls. However, this study only involved n = 15 SS patients, and an equivalent number of controls. As expected, SFR was found to be significantly lower in the Sjorgen’s syndrome participants than in the control group (0.82 ± 0.29 versus 1.99 ± 0.51 mL/min, respectively).

From this work, the authors concluded that high-resolution ^1^H NMR spectroscopy offers much potential for the quantitative metabolic profiling of human saliva specimens. It was also concluded that NMR analysis is suitable for the analysis of neurotransmitter amino acids (NAAs, specifically glycine, glutamate and γ-aminoglutamate (GABA) in human saliva), and that it may by-pass other analytical protocols and techniques which are only able to analyse only single or small numbers of biomolecules.

However, without allowing for possible contributions from an enhanced activity level of bacterial-mediated proteolytic damage to salivary proteins, how can these be distinguished from those arising from the SS disease process itself? In any case, we observed, confirmed and validated the successful ^1^H NMR-based detection and analysis of NAAs in WMSSs by means of 1D and 2D NMR techniques over 20 years ago [18]. Similarly, levels of the excitatory amino acid, such as aspartate, were also readily determined using this technique (ABX coupling pattern: δ = 2.69/2.79 -CH_2_ (AB) and 3.89 ppm -CH (X)).

In a related study, this same research group explored the variation is salivary biomolecule concentrations and outputs in SS patients and age-matched healthy controls (n = 14 and 15 participants, respectively); these participants donated single saliva sample only once per week for a 4-week period [66]. However, the method that they employed for evaluating ‘within-participant’ variability is questionable, since the results appear to be analysed with sample collection week as a ‘fixed’ and not a ‘random’ effect, which indeed it is. Moreover, there was no partitioning of total variance into ‘between-’ and ‘within-’ components as demonstrated in Section 10.1.1 of Part I of this series of papers [1]. However, the results acquired indicated that choline, taurine, alanine and glycine had significantly upregulated salivary levels in SS patients, but for all assessment weeks involved, this was only the case for choline; clearly, the above recommended isolation of all sources of variation, both fixed and random, in an experimental design of experiments model would have served to clarify results obtained on this dataset. However, in general, inter-participant variations in metabolite levels was found to be greater amongst Sjögren’s patients than the controls, and significantly high variabilities were noted for glycine, choline and alanine. However, choline had the lowest ‘within-participant-between-weeks variation’. From this work, the authors concluded that salivary levels and outputs (the latter represented by the product of SFR and salivary concentration) of selected biomolecules may, at least in principle, be employed as new biomarkers for primary SS, irrespective of a considerable level of between- and within-patient variation.

Further non-NMR-based metabolomics research focused in this research area include an examination of the high fidelity between salivary proteomics data and the biological status of the salivary glands, which provides valuable biomarker patterns for primary SS [62]; the identification of genomic biomarkers for this condition employing an approach featuring the pooling of gene expression microarray data [63]; and a preclinical validation of both protein and mRNA transcript biomarkers for primary SS, which may distinguish between these patients and those with systemic lupus erythematosus, and healthy controls [64].

Finally, for SS patients, and others with conditions which also feature limited salivary availability and/or dry mouth characteristics, a further complication is that NMR analysis performed on most high-field spectrometers available requires a relatively large saliva sample (ca. 0.5 mL). Indeed, this collection process may be rather demanding regarding the donation of unstimulated, WMS samples from patients with xerostomia. Unfortunately, in some studies NMR investigations were not originally planned for metabolomics investigations, and therefore the provision of sufficient volumes of samples has not been certified systematically. Therefore, the embodiment of careful experimental designs, with acceptable and pre-planned provisions for some patient cases featuring the donation of only insufficient sample volumes, are required. Notwithstanding, in view of the availability of 3 mm diameter NMR tubes, for which volumes as low as 0.10 mL may be used for analysis.

Interestingly, one further recent study [67] reported that a low-dose doxycycline (LDD) therapy normalises the salivary concentrations of some metabolites in primary SS patients. In the protocol followed, stimulated saliva specimens were collected from groups of these patients (all female), the first being treated with LDD, the second being untreated with this therapy. A third group comprising a healthy age-matched female control group also donated these biofluid samples. The metabolic profiles of these samples were subsequently analysed using an LC/MS technique based on an untargeted metabolomics strategy, and the salivary biomolecular patterns found for each of these three groups were then compared and contrasted. Of especial interest, the untreated primary SS group displayed a metabolic signature which was distinct from that of the healthy controls. Moreover, salivary levels of a series of biomolecules, including the tyrosine-glutamine, phenylalanine-isoleucine and valine-leucine dipeptides, phenylalanine, the cholesterol ester cholesteryl palmitate, pantothenate (vitamin B5) and urocanate biomarkers were returned to their ‘normal’ healthy control values following LDD therapy. The authors concluded that a deviating salivary metabolic pattern was observed in primary SS patients, and that treatment with LLD effectively normalised the concentrations of metabolites related to oral microbiota dysbiosis in these patients.

### 4.5. Neurological Conditions

#### 4.5.1. Parkinson’s Disease

Parkinson’s disease (PaD) represents a multisystem disorder of unknown aetiology, which displays a broad profile of symptoms, along with pathological attributes which affect organs throughout the human body. Indeed, in principle, the metabolic profiles of WMS collected from PaD patients may be altered by malabsorption in the gastroenteric tract, neurodegenerative processes and mitochondrial disturbances. Very recently, Kumari et al. [68] employed an NMR-based metabolomics strategy for the identification and evaluation of biomarkers for PaD in saliva samples collected from a total of n = 76 patients with this disorder; results acquired were compared with the salivary ^1^H NMR profiles of n = 37 healthy controls. In this investigation, upregulated levels of aromatic amino acids (phenylalanine, tyrosine and histidine), glycine, acetoacetate, taurine, trimethylamine-N-oxide, γ-aminobutyrate, N-acetylglutamate, acetoin, acetate, alanine, fucose, propionate, isoleucine, and valine were found in PaD patients when normalised to those of the healthy control participants. Moreover, an additional sub-group examination demonstrated increased metabolite concentrations in an early PaD group over those of advanced PaD and healthy control classifications. Results acquired from these experiments suggested that PaD patients could be articulated by metabolic disturbances in energy and gut microflora systems, together with neurotransmitters (e.g., glutamate, glycine and γ-aminobutyrate), which possibly augment the pathogenesis of PaD. However, salivary upregulations seen for metabolites arising from the salivary microbiome (propionate, acetate and acetoin, plus at least some amino acids) may simply arise from poor or less vigorous oral healthcare regimens associated with PaD patients, i.e., less frequent tooth-brushing and oral rinsing episodes, etc.

#### 4.5.2. Alzheimer’s Disease and Age-Related Dementia

Similarly, the metabolic status of saliva collected from patients with Alzheimer’s disease was investigated by Yilmaz et al. (2017) [69], and they observed that a combination of propionate and acetone provided a metabolic signature for this condition. Furthermore, propionate was also observed to be upregulated in patients with dementia when compared to a control cohort, in addition to increased concentrations of acetate, and apparently histamine in a very large and extensive investigation conducted by Figueria et al., 2016 [70]. Significantly lower salivary levels of taurine, succinate, glycerol and dimethylsulphone were also observed in dementia patients. These dementia patients had either Alzheimer’s disease or vascular dementia at the primary sampling time, or they subsequently developed one of these conditions before a pre-set follow-up sampling/evaluation episode scheduled five years later (an age-, gender-, and education-matched control cohort of participants without dementia were also recruited). From this study, the researchers involved surmised that some of these biomarkers were directly implicated in the pathogenesis of Alzheimer’s disease (e.g., acetate, glycerol, histamine, succinate and taurine), whereas others, such as propionate, reflected the poorer periodontal status of ageing patients with dementia [70].

However, a close inspection of the ^1^H NMR profiles acquired on both unfiltered and filtered saliva samples reveals a number of analysis and interpretational flaws, along with further problems. Firstly, salivary histamine concentrations are simply not sufficiently high enough for ^1^H NMR detection at an operating frequency of 600 MHz; indeed, such mean salivary levels in heathy controls are predominantly reported as sub-micromolar (ca. 100–300 nmol./L), with the exception of one study which reported ca. 3 μmol./L [42], and for samples featured in this latter investigation, histamine was determined via a direct flow injection (DFI)/LC–MS/MS technique and not by ^1^H NMR analysis. However, in principle, higher levels of salivary histamine are possible following the collection of samples immediately following a histamine-rich meal.

Moreover, despite being assured that the ultrafiltration devices employed for this study were thoroughly pre-cleansed, it is possible or even probable that the glycerol found in the ultrafiltered WMS samples analysed arises from the use of such facilities, an artefact previously observed by the authors, for example when employing those with a 3 kDa cut-off value. Therefore, without careful laboratory housekeeping and planning, and steps taken to circumvent such problems (including the removal of chemical shift buckets containing ultrafiltration device-derived agents such as glycerol prior to MV dataset analysis), the laboratory research use of such devices can easily introduce such sample artefacts, a process which will possibly render all further metabolomics data generated completely invalid.

### 4.6. Chronic Apical Abscess

Chronic apical abscess (CAA) represents an apical periodontitis lesion which is mainly manifested by regions of liquefactive necrosis with deteriorating polymorphonuclear neutrophils (PMNLs) enveloped by macrophages. This environment gives rise to local bacterial infection and a systemic inflammatory response, along with swelling and pain. Recently, Montis et al. [71] applied metabolomics approaches for the discovery of biomarkers which provide some novel molecular fingerprinting discernments regarding the pathogenesis of CAA via the multianalyte analysis of human saliva samples using GC/MS technologies. This study involved n = 11 CAA patients, and n = 11 controls without any clinical or radiographic signs of CAA. Overall, a MV statistical model was constructed which effectively distinguished between these two groups of participants, and the classes of biomolecules characterising the CAA salivary profiles were found to arise from tissue necrosis, bacterial catabolism and sinus tract activities. These preliminary results may be valuable for the diagnosis of CAA, and may also provide useful biomolecular information regarding its pathological basis, although the small sample size involved will limit such applications.

### 4.7. Respiratory Diseases

The association between the status of the oral cavity and respiratory diseases was recently assessed by Imai et al. [72] with the objective that lower airway inflammation may be influenced via the aspiration of oral bacteria. With an increasingly ageing global population, there is now an increasing incidence of lower airway diseases. Indeed, the elderly population have diminished capacities in swallowing function and airway ciliary flexibility, together with an ageing-related overall immunity. Additionally, since such lower airway diseases in the elderly population have an associated high mortality rate, the development of methods for their prevention will serve to improve the longevity and quality of life of subjects affected.

This report [72] outlines and discusses the relevance of now proven associations between chronic periodontitis (CD), pneumonia and chronic obstructive pulmonary disease (COPD), all of which have been reported in a significant number of studies. Useful information on mechanisms for the development, progression and/or exacerbation of these airway diseases and oral bacterial pathogenicity are provided. The authors also evaluated the roles of oral bacteria in the spread of influenza infection, and the aggravation of infectious diseases in general. Relationships between oral flora constitution and the severity of SARS-CoV-2 infection was also explored.

Therefore, there appear to be ample opportunities for the metabolomics analysis, ^1^H NMR-based or otherwise, of human saliva, or more specifically those focussed on selected catabolites arising from oral microbiome sources [1], for the delineation of these adverse associations. Indeed, any validated biomarkers identified from such investigations may, at least in principle, be indirectly employed for the diagnosis of these airway diseases, and for probing their development, progression and aggravation in the human population.

### 4.8. Viral Infections

#### 4.8.1. General Overview of the Influence of Viral Infections on Human Host Metabolism

To date, there has been a very high level of interest in determining the interactions of viruses with host metabolic pathways, and a number of investigations have reported the mechanisms by which viral infections may exert effects on those involving, for example, glucose, FAs, nucleotides and proteins. Interestingly, Girdhar et al. [73] explored these effects, albeit focussing on host metabolic transformations of glucose, FAs and glutamine, and for further reading they have drawn attention to those pathways which are exploited by different types of viral species in order to enhance their replication. Moreover, they have also delineated and reviewed routes by which the host endocrine system is adopted and used by selected viral hormones. Notably, these researchers discuss viral insulin/insulin-like growth factor 1 (IGF-1)-like peptides and their ability to affect host metabolism. Although there are only limited numbers of reports available on this topic, the synchronisation of metabolic pathways in the human host represents a major factor of viral durability and replication. However, to date, there is only limited information available on the ability of different viral species to focus on specific pathways. Viruses considered have the ability to influence a range of signaling pathways in order to modify biomolecule metabolism, and one prime example being the phosphoinositide 3-kinase (PI3K)/AMP-activated protein kinase (AMPK)/mammalian target of rapamycin (mTOR) signaling system to control glucose metabolism, this effect occurring irrespective of viral types involved and their hosts. Moreover, selected transcription factors, e.g., Myc, sterol regulatory-element binding protein (SREBP), and hypoxia-inducible factor (HIF)-1α are virally activated to facilitate virus replication and longevity within host cells. Notably, the inhibition of such signalling pathways, including key catalytic enzymes, has been demonstrated to block viral replication, both in vitro and in vivo. Additionally, recently discovered viral insulin-like peptides (VILPs) offer novel routes towards host-pathogen associations in which the hormones which directly regulate host metabolism are closely imitated by viruses. Further validated studies focused on these effects and their associated mechanisms will undoubtedly improve our understanding of viral pathogenesis; such developments may also shed light on the design and development of new therapies for the treatment or circumvention of viral infections in humans.

#### 4.8.2. Influenza

Previously conducted metabolomics investigations of influenza infection have been found to influence a wide range of cellular metabolic pathways in order to optimise the development of suitable environments for viral particle replication and generation (reviewed in [74]). Indeed, subsequent to infection, the rate and extent of glucose uptake and aerobic glycolysis continuously rise in infected cells, a phenomenon giving rise to an enhanced level of glucose consumption by them. Likewise, a further glucose-devouring pathway, the pentose phosphate shunt, is also upregulated by influenza infection to facilitate nucleotide production, notably that of ATP. Infection-mediated disturbances to triacylglycerol, phospholipid and secondary lipid adducts have also been observed, and a number of these are linked to inflammatory responses. Concomitantly, the β-oxidation of mitochondrial FAs diminishes significantly, with a corresponding enhancement in FA and membrane lipid biosynthesis. Furthermore, downregulations in essential amino acids have also been found in infected tissues, and this process is generally triggered by the generation of high levels of both viral and cellular proteins. However, it should also be noted that the augmentation of immune responses against influenza infection may significantly influence and disrupt metabolic pathways. Overall, influenza viral infection-mediated interferon (IFN) formation exerts a major outcome on cellular function via modifications in lipid metabolism, lipid membrane status and amino acid biosynthesis. Therefore, as in our study regarding the predominantly viral infection of humans afflicted by an acute sore throat condition, an increased understanding of host metabolic perturbations mandatory for influenza virus replication has, to date, demonstrated that the targeted suppression of these pathway modifications may yield valuable information regarding the design, development and management of new therapeutic agents and regimens for the treatment of this condition.

Moreover, influenza virus neuraminidase (NA) has the ability to cleave N-acetylneuraminate (sialate) residues located at the termini of the molecularly mobile glycoprotein oligosaccharide chains that serve as virus binding receptors. In this manner, one important action for them is the release of virions from infected cells, a process aiding the proliferation of cell-to-cell infection. Moreover, this enzyme acts at the primary phase of viral infection within the respiratory tract via the deterioration of inhibitors of hemagglutination in biofluids, and which emulously block virus receptor binding. First-line anti-influenza drugs comprise specific inhibitors of viral NA, although they do not also inhibit this class of enzymes in bacteria. In 2012, Nishikawa et al. [75] explored that capacity of bacterial neuraminidase to ‘rescue’ the replication of influenza virus from inhibition exerted by a neuraminidase inhibitor. Indeed, the authors of this report hypothesised that in view of the knowledge that oral and upper respiratory commensal bacterial flora contain those with the ability to generate NAs, such enzymes could restrict the anti-viral activities of NA inhibitor drugs within respiratory organs during inhibition of viral NA. Therefore, they employed in vitro models of infection in order to explore the actions of bacterial NAs on influenza virus infectivity when the NA inhibitor drug zanamivir is available. Indeed, it was observed that zanamivir diminished the yield of progeny virus to <2% of that found in controls, although this process was predominantly restored via exogenous treatment with bacterial *Streptococcus pneumoniae* neuraminidase. Additionally, cell-to-cell infection was markedly hampered by zanamivir, but was reimposed through bacterial neuraminidase addition. The influence of bacterial NA on the suppression of hemagglutination activity and inhibition of the infectivity of human saliva was then investigated with added zanamivir, and the investigators found that whilst this drug stimulated both sets of salivary inhibitory properties, bacterial NA significantly suppressed this augmentation. The authors concluded that their results demonstrated that bacterial neuraminidases (NAs) served as the predominant NAs for the inhibition of viral NA, and in this manner facilitated the spread of infection, along with deactivation of the viral terminating activity of human saliva. Therefore, the authors proposed that bacterial flora neuraminidase may impede NA inhibitor drug activities throughout influenza virus infection in humans.

#### 4.8.3. HIV Infection

Novel salivary biomarkers may provide accessible avenues for tracking the development of HIV infection to full-scale acquired immune deficiency syndrome (AIDS), and for evaluating patient responses to therapeutic strategies administered. Indeed, NMR- and LC/MS- based analytical approaches have to date proven to be valuable methodologies for driving the assessment of phenotypic modifications in infected subjects, and has already served to recognise chemopathological routes and metabolic pathways associated with its invasion and infiltration of biofluids, organs and tissues. Notably, both these bioanalytical techniques have shown that this retrovirus affects carbohydrate and lipid metabolism, which indicates that highly specific metabolic imbalances are induced by HIV infection. Moreover, highly active antiretroviral treatment (HAART) also exerts a significant influence on these two major routes (reviewed in [76]). A limited number of studies have served to discriminate between HIV-negative, -positive and treatment-responding individuals, and have also defined putative biomarkers. However, there remains a myriad of experimental problems with data acquired. Firstly, these metabolomics data are predominantly only qualitative; secondly, assignments for the identities of these markers and other biomolecules have not always been validated, and nor have the MV statistical strategies employed for testing for differences between groups; and thirdly, the attribution of selected metabolic modifications to the therapeutic benefits offered by drug treatments using metabolomics analysis requires much further investigation in any case. This review also comments on metabolites routinely detected as being affected by the pathogen or treatment, explains what existing data suggests, and additionally makes recommendations on future research tasks to be conducted.

In 2013, Munshi et al. [77] explored modifications to the metabolic profiles of blood plasma, urine, and saliva specimens collected from a cohort of HIV/AIDS patients, including those receiving anti-retroviral therapy (ART). In this study, PLS-DA was employed for the generation of three principal component models, which demonstrated that plasma and urine were more effective than saliva in distinguishing between HIV/AIDS patients and healthy controls, and between ART-receiving patients and those not receiving this treatment. A total of 26 biomolecules were found to be significantly influenced in all or two classes of biofluid samples. The authors indicated that blood plasma choline and sarcosine, along with urinary neopterin, may be employed as biomarkers of HIV/AIDS infection.

#### 4.8.4. SARS-CoV-2 Infection

In view of the continuous emergence of the COVID-19 pathogen as a major global public health threat, many metabolomics studies, notably but not exclusively those based on NMR analysis, have been performed during the last year or so in order to design, develop, validate and operate reliable diagnostic tests for its human infection, and to take steps to understand mechanisms and pathways underlying its pathological actions. Indeed, metabolomics and metabolomics-related strategies serve as extremely valuable tools for this purpose, and for the prognostic screening of the course of disease induced by SARS-CoV-2 infection. Therefore, explorations of alterations in the salivary metabolic profiles of participants or patients following COVID-19 infection may provide valuable information relevant to the monitoring of disease progression, as well as the heterogenous natures of its clinical phenotypes, as recently proffered by Costa Dos Santos et al. [78]. Together with minimally invasive sample collection regimens, saliva offers further advantages as a COVID-19 testing matrix, since this virus raids and infiltrates oral mucosal epithelial cells and salivary gland ducts through its angiotensin-converting enzyme (ACE)-2 receptors. In fact, Xu et al. [79] describe salivary glands as potential reservoirs for COVID-19. Therefore, SARS-CoV-2 salivary metabolic fingerprinting may provide valuable information on our understanding of the pathophysiology of its infection, most notably if studies are carefully designed and samples are longitudinally monitored.

Through a detailed literature review, the paper by Marques et al. [80] analysed evidence available for oral cavity manifestations of COVD-19, in order to deduce whether they primarily arise from the virus itself, or from secondary phenomena. For this objective, a narrative review of papers collected from four defined search platforms was conducted (only published articles from 2020 were selected; following refinement, 24 papers were selected for consideration). Of the oral cavity manifestations recorded, taste changes were found to occur in the majority of cases. Moreover, in a number of the documented articles examined, vesiculobullous and ulcerative oral lesions were observed, although the authors concluded that additional investigations should be conducted to confirm these manifestations, most especially because of the limited amount of information available at the time of publication. Actually, the new emergence of COVID-19 infection was provided as a further reason for these sources of conjecture at that point in time.

Such oral cavity manifestations may be caused or reflected by COVID-19-induced changes in the salivary oral metabolome, and therefore such agents may serve as useful biomarkers for a positive infection with this virus, and its associated oral developments.

#### 4.8.5. Pharyngitis (Acute Sore Throat Conditions)

Sore throat, which is also known as ‘pharyngitis’ or ‘tonsilitis’, is an infection of the acute upper respiratory tract which influences the throat’s respiratory mucosa. Moreover, it is also linked to headache, fever and general malaise. Additional suppurative complications of this condition are acute sinusitis, peritonsillar abscess and acute otitis media, most notably the latter. Of all adult acute sore throat conditions, approximately 90% of adult sore throat disease cases result from virus infections, as indeed are approximately 70% of those occurring in children (aged 5–16 years) [81]. Those remaining are ascribable to bacterial infections, particularly group A β-haemolytic streptococcus (GABHS). Sore throat conditions derived from GABHS are characterised and clinically identified through the observation of augmented submandibular glands and rhinorrhoea (runny nose), together with the presence of a throat exudate, although these signs and symptoms are not accompanied by cough and fever [81,82]. Fortunately, sore throat conditions caused by viruses are commonly self-resolving, whereas those attributable to bacterial infections generally require antibiotic treatment.

In a quite recent study, our group linked a newly developed fuzzy genetic programming-base for a subgroup discovery (FuGePSD) algorithmic computational intelligence technique for the analysis and interpretation of results acquired from the high-field ^1^H NMR analysis of human saliva [83] performed to investigate biomolecular pathological mechanisms of acute sore throat conditions in humans. This metabolomics study was focused on the recognition of quantitative patterns, and directional routes, of salivary biomolecules which may be characteristic of viral- (and possibly bacterial)-triggered forms of this disorder in humans. Therefore, the ^1^H NMR spectral patterns of healthy and acute sore throat condition participants were analysed using the FuGePSD strategy in an experiment involving >200 intelligently selected bucket (ISB) ^1^H NMR predictor variables, and almost 500 samples (5 replicated daily saliva samples donated by a grand total of 96 participants).

Application of the FuGePSD strategy resulted in the segregation of HSS samples into 5 sub-groups, four characteristic of active sore throat diseases, and a single one corresponding to healthy control participants. Indeed, the FuGePSD technique had the ability to optimize the classification of sub-groups with only a small number of predictor biomarker variables in order to describe the target sub-group, which highlight predictor variable values in the context of their ‘unusualness’ and ‘trade-off’ sensitivity confidence [83]. Generally, sub-group distinctiveness was precise, with a mean confidence of 86%. Moreover, the effectiveness of this sub-group classification algorithm was as high as 95%.

Valuable salivary biomarker features detected for the single healthy control sub-group were trace protein levels, including those with ^1^H NMR-visible tyrosine residue resonances (Figure 1), acetoin and glycine. However, those for the four sore throat disease sub-groups featured 5-aminopentanoate and aspartate [83]. The identities of the 5-aminopentanoate signals were verified from an examination of the 2D COSY ^1^H/^1^H–^1^H NMR profiles of the WMSS samples. Specifically, it was observed that the 2.24 ppm resonance was closely correlated to those located at δ = 1.66 (two sets of overlapping *tt* multiplets), and 3.025 ppm (*t*) of relative intensities 2.0 and 1.0, respectively when normalised to that of the 2.24 ppm resonance—these signals were clearly assignable to 5-aminopentanoate’s 3-/4- and 5-position -CH_2_- group protons, respectively (that at δ = 2.24 ppm was attributable to the *α*-CH_2_- (2-position) protons of this biomolecule) [1].

**Figure 3:**
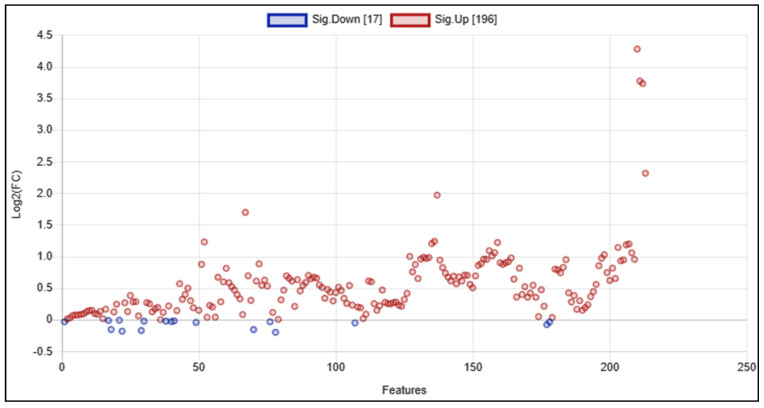
Significantly up- and downregulated values for univariate log_2_ fold-changes computed from WMSS sample ISB buckets for the comparison of patients with an acute sore throat condition with those of an age-matched healthy control group (n = 48 participants per group, each one providing n = 5 sequential daily samples).

A very high percentage (92%) of the TSP-normalised ^1^H NMR ISB disease-determining predictor variables were found to be of a higher or much higher intensity in the active sore throat disease cohort of participants than they were in the healthy control group (Figure 3). Importantly, this observation may at least partially be ascribable to dehydration in sore throat participants with associated lowered SFR values, a phenomenon which serves as a common sign or symptom of this disorder, as indeed it is for patients with Sjörgen’s syndrome.

5-Aminopentanoate, which is upregulated in participants with sore throat conditions, and is a microbial metabolite generated from the bacterial catabolism of L-lysine [84], has been shown to be biosynthesised from cadaverine in mice [85], although it may also be generated from endogenous (host) sources in humans [1,56]. Hence, its upregulation in humans with acute sore throat disease may correspond to an increased microbial growth level in the oral environment of such patients. Additionally, sore throat condition-induced upregulations in salivary acetoin may arise from fermentation processes involving the microbial butanediol cycle.

## 5. Original Case Study: Further Investigations of the NMR-Based Diagnosis and Pathogenesis of Acute Sore Throat Conditions in Humans

### 5.1. Introduction

In this case study, we applied ^1^H NMR-linked metabolomics analysis in order to seek and recognise salivary biomolecule patterns which are potentially characteristic of viral- (and bacterial-, if also relevant)-induced acute sore throat (pharyngitis) disorders in humans. This involved MV comparisons of the ^1^H NMR spectra of this biofluid in patients afflicted by this disease with those of healthy, non-medically compromised controls. All participants provided verbal or written informed consent for participation in the investigation. This study excluded participants who had been taking any type of medication during the 7 days prior to the very first sampling date. Participants were also requested not to receive any form of medication throughout the 5-day duration of the non-interventional trial.

Therefore, the experimental design involved encompassed an experimental design model with n = 48 participants classified within either of the two groups. All recruited participant provided WMS samples once daily for a sequential 5-weekday period. This model was employed to explore the diagnostic capacity and overall value of each biomolecule monitored as ^1^H NMR ISBs. According to our WMS collection protocol outlined in Ref. [1], all participants were instructed to collect mouth-expectorated WMS samples into a plastic sterile universal tube immediately after waking in the morning on each consecutive trial day. All participants were also requested not to engage in any type of oral activities (including drinking, eating tooth-brushing, oral rinsing, cigarette smoking, etc.) during the 5 min or so duration between awakening and WMS collection.

### 5.2. Transport, Preparation and Storage of WMS Samples Prior to ^1^H NMR Analysis

WMS samples were transported to the laboratory on ice, and then centrifuged immediately (3500 r.p.m. for 15 min) on their arrival to in order to separate and discard cells and debris, and supernatants derived therefrom (WMSSs) were then stored in a laboratory deep freezer facility at a temperature of −80 °C for a maximal period of 72 h prior to the performance of ^1^H NMR spectroscopic analysis. Each WMSS specimen was then treated with sodium fluoride (15 μmol.) to prevent the enzymatic generation of microbial catabolites therein throughout the required transport, sample preparation and/or storage durations.

Pre-fixed aliquots (0.60 mL) of WMSS samples were then transferred to 5 mm diameter NMR tubes, and a 0.10 mL volume of a 2.50 × 10^−3^ mol.dm^−3^ solution of TSP (internal chemical shift reference, δ = 0.00 ppm and internal quantitative ^1^H NMR standard) in deuterium oxide (^2^H_2_O) was added, the latter serving as a field frequency lock.

### 5.3. Acquisition of 600 MHz ^1^H NMR Spectra of WMS Supernatant Samples

**Figure 4:**
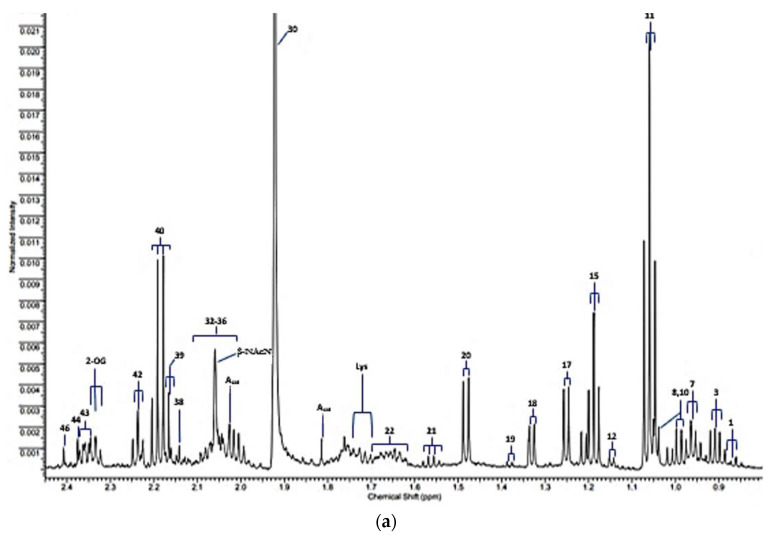

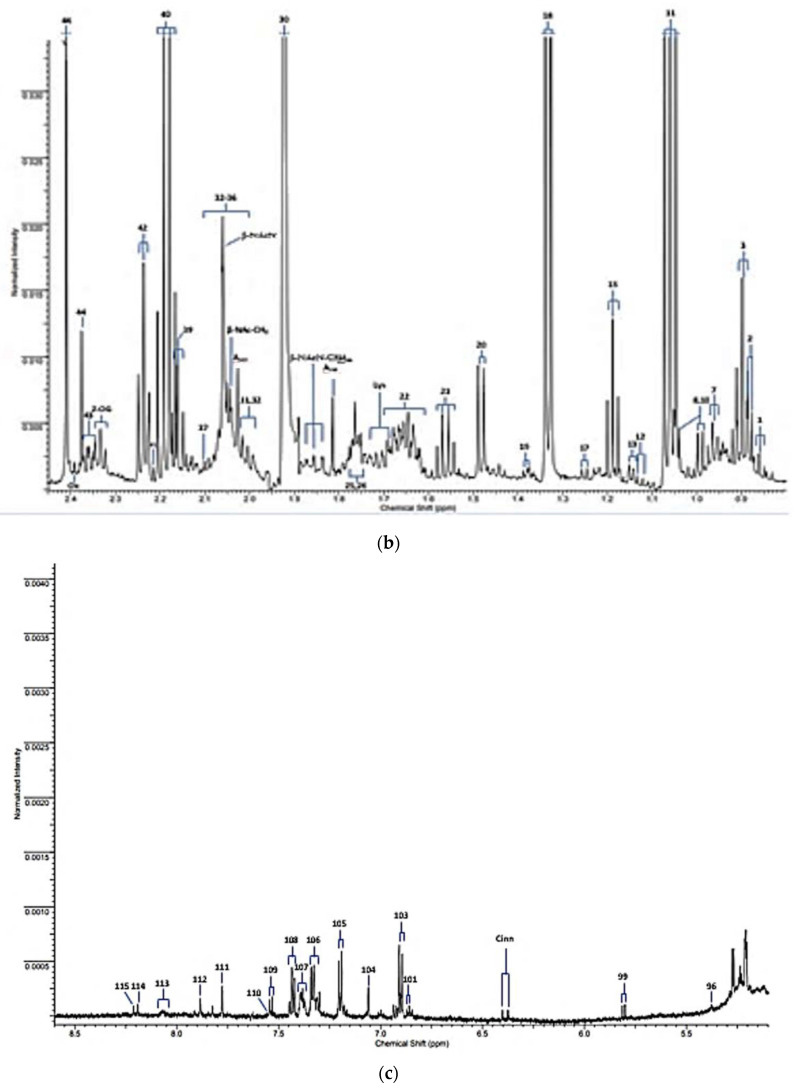

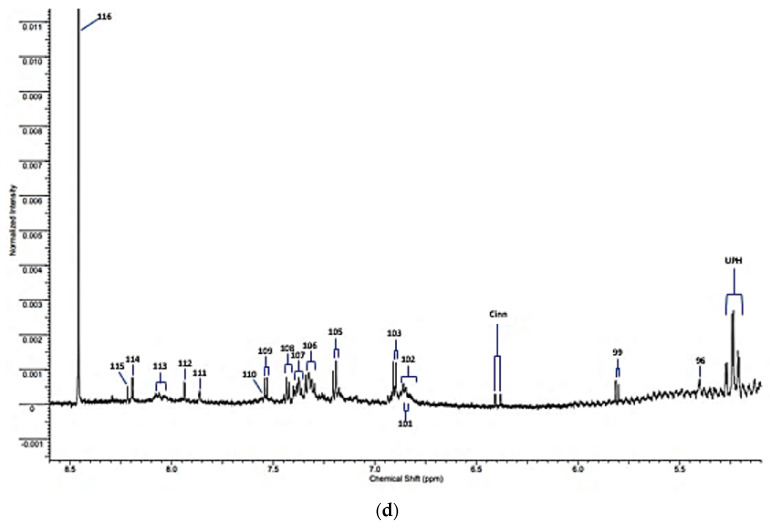
(**a**,**b**) 0.80–2.45 ppm regions of the 600 MHz single-pulse *noesy-presat* ^1^H NMR profiles of WMS supernatant samples donated by healthy and acute sore throat disease human participants, respectively. (**c**,**d**) Corresponding 5.10–8.60 ppm regions of these spectra, respectively. Typical spectra are shown. Abbreviations: Assignment codes correspond to those listed in Table 1, along with a small number of tentative assignments. Spectra were acquired as described in Section 5.3 above.

Pre-optimised single-pulse *noesy-presat* ^1^H NMR spectra were obtained on a Bruker Avance AM-600 spectrometer (Bruker UK Ltd, Coventry, UK), which was operating at a frequency of 600.13 MHz, in quadrature detection mode and a probe temperature of 293 K (acquisitional pulsing conditions and parameters employed are provided in the legend to Figure 1 above). Both the high- and low-field regions of 600 MHz *nosey-presat* ^1^H NMR spectra acquired on typical WMSS samples derived from WMS samples donated by both healthy control and acute sore throat disease participants, are shown in Figure 4.

### 5.4. Preprocessing of Salivary ^1^H NMR Spectral Profiles, and Statistical Analyses of MV Datasets Arising Therefrom

Salivary ISB intensities were normalised to that of an added TSP internal standard. ^1^H NMR data matrices (480 spectra × 209 ISB buckets, or 480 spectra × 31 assigned named metabolites) were produced via the employment of macro processes for line-broadening, zero-filling, Fourier-transformation, and phase and baseline corrections. This was followed by the application of a separate macro system for the intelligent bucketing of spectral resonances. All unwanted interfering resonances (exclusively that of the highly intense H2O/HOD at δ = 4.8 ppm), together with all spectral ‘noise’ regions, were removed from the spectral profiles acquired prior to performing the bucketing process. These computations were executed using the ACD/Labs 1D NMR Manager software package (Advanced Chemistry Development, Inc. (ACD/Labs), Toronto, ON, Canada).

MV data analysis was then performed using *MetaboAnalayst 5.0* software modules. This software approach was largely employed to explore the applications of PLS-DA and sparse PLS-DA (sPLS-DA) analyses of the large dataset acquired, and to determine the differential effects of row-wise probabilistic quotient and constant sum normalisation approaches (PQN and CSN, respectively) on the results acquired. Hence, where imputed, datasets were row-wise normalised using either of these approaches, the latter involving expression of all ISB intensities to that of an average ^1^H NMR spectral profile computed from the H NMR profiles of all healthy control group WMSS samples acquired. Both these normalisation methods serve as a means of overcoming problems with dehydration-mediated increases in the salivary concentrations of metabolites in the active sore throat group of participants. These further row-wise normalisation strategies were applied subsequent to the performance of the TSP normalisation process. All datasets were then generalised logarithmically (glog-) transformed and Pareto-scaled prior to data analysis.

A random forest (RF) model option was also applied using this MV analysis module, using 1000 tress and 7 variables per node. This involved a model featuring TSP-normalisation, glog-transformation and Pareto-scaling, but without any further row-wise normalisation technique applied.

Quantitative pathway topological and metabolite enrichment analysis was also conducted using *MetaboAnalayst 5.0* software modules. For this purpose, datasets were PQN-normalised, glog-transformed and Pareto-scaled prior to analysis. Full details regarding the algorithms and analysis strategies employed for this purpose are provided in Section 5.6 below.

### 5.5. Multivariate Metabolomics Analysis of ^1^H NMR Datasets

For this investigation, primarily, we performed PLS-DA and RF approaches for the MV analysis of these sore throat study data, and this was conducted firstly by either using all 31 assigned, named biomolecules identified by ^1^H NMR analysis, or the complete dataset with 183 ^1^H NMR ISBs included as potential predictor variables (Model 1). Secondly, we incorporated only those loading significantly on the PC deduced to arise from the host metabolite source (PC1), and not that from the oral microbiome (PC2), as outlined in Part I of this series of papers [1] (Model 2). As noted below, justification for this was provided by the observation that the majority of the key distinguishing biomarkers found from MV analysis of the Model 1 datasets were mainly derived from the host (PC1) source.

Primary analysis involving all 31 named analyte variables (i.e., those contributing significantly towards all PCs (PCs 1–4)), with internal standard TSP-normalisation as the only row-wise normalisation strategy, and with glog-transformation and Pareto-scaling processing, revealed only a marginal level of distinction between the clinically established pharyngitis and age-matched healthy control groups (data not shown). Although the maximal Q^2^ value obtained was only 0.19, a permutation test conducted with 2000 permutations was very highly significant indeed (*p* < 5.0 × 10^−4^), and hence this confirmed a significant, albeit still poorly predictive, level of distinctiveness between these two disease classifications. Key biomarkers found, i.e., those with variable importance parameter (VIP) values > 0.90, were glutamate (↑) > glutamine (↓) > pyruvate (↓) > urea (↑) > 3-D-hydroxybutyrate (↓) > alanine (↓) > taurine (↓) > phenylalanine (↓) > succinate (↓), the arrows representing up- or downregulations in the sore throat-positive group. Notably, the top four significant predictor variables predominantly arise from the host and not the oral microbiome source. Indeed, their loading contributions towards the oral microbiome-sourced principal component (PC2) are quite small for each of these biomolecules [1], with loadings vectors of only 0.26 (glutamate) 0.35 (glutamine) 0.47 (pyruvate) and 0.23 (urea) using a threshold loading significance value set at 0.35 [1]. The elevated PC2 contribution for pyruvate is explicable by its dual microbial and host sources, the former occurring through its catabolic restriction in such species; indeed, this metabolite represents a major metabolic excretion product in some bacteria [86]. More notably, termination of the pyruvate-acetate efflux in bacteria was found to give rise to the build-up of a pyruvate-induced acid resistance. Indeed, this pyruvate-dependent resistance was not restricted to glucose-supplemented bacteria, but was also fully functional in those grown on a range of sugar fuels [87].

**Figure 5:**
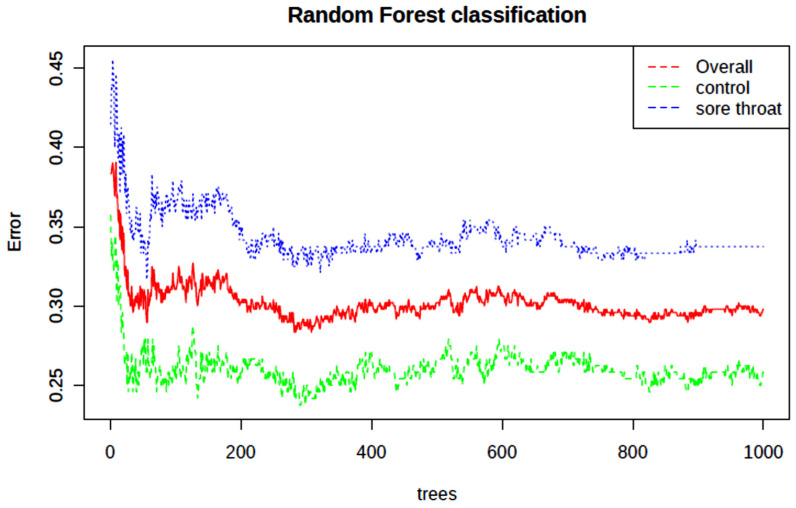
Random Forest plot of classification error versus number of trees incorporated into the analysis (Model 1) which was performed on all 31 named, assigned metabolites available. This dataset underwent TSP-normalisation, glog-transformation and Pareto-scaling prior to analysis, but without any further row-wise normalisation applied.

**Figure 6:**
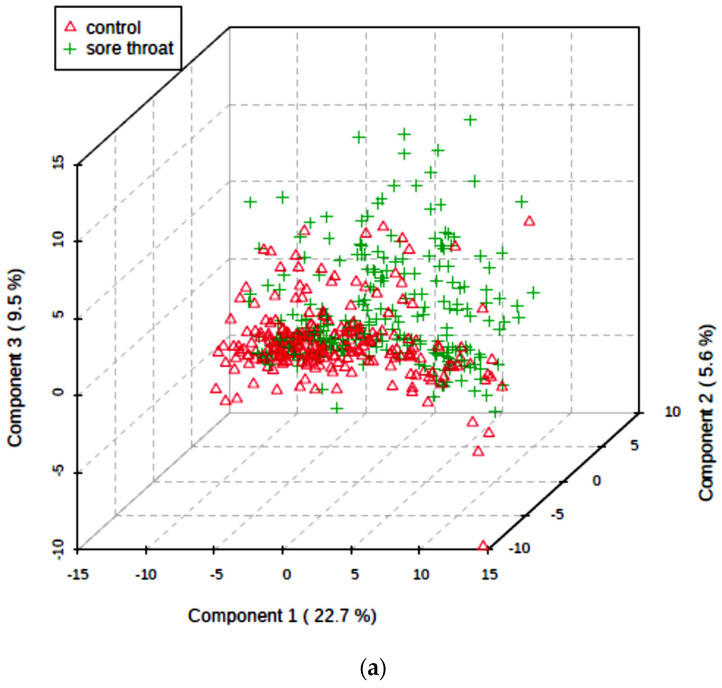

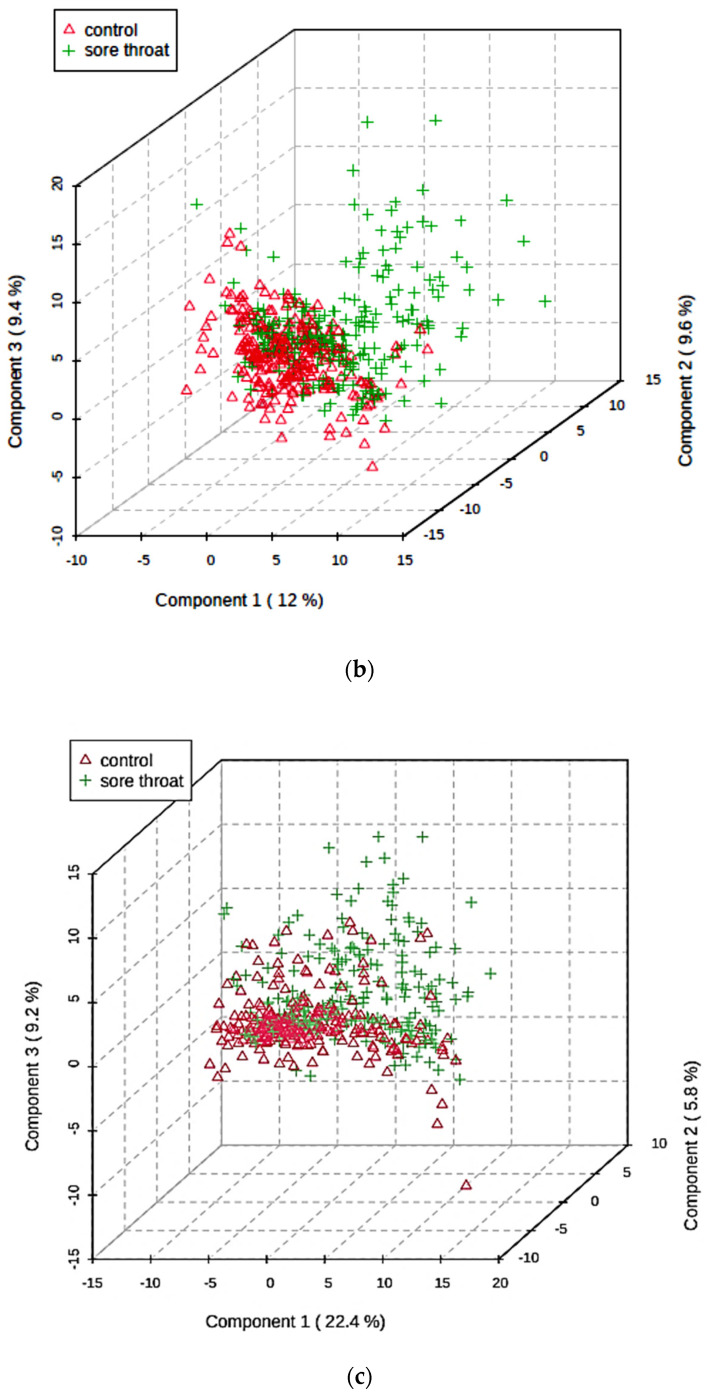

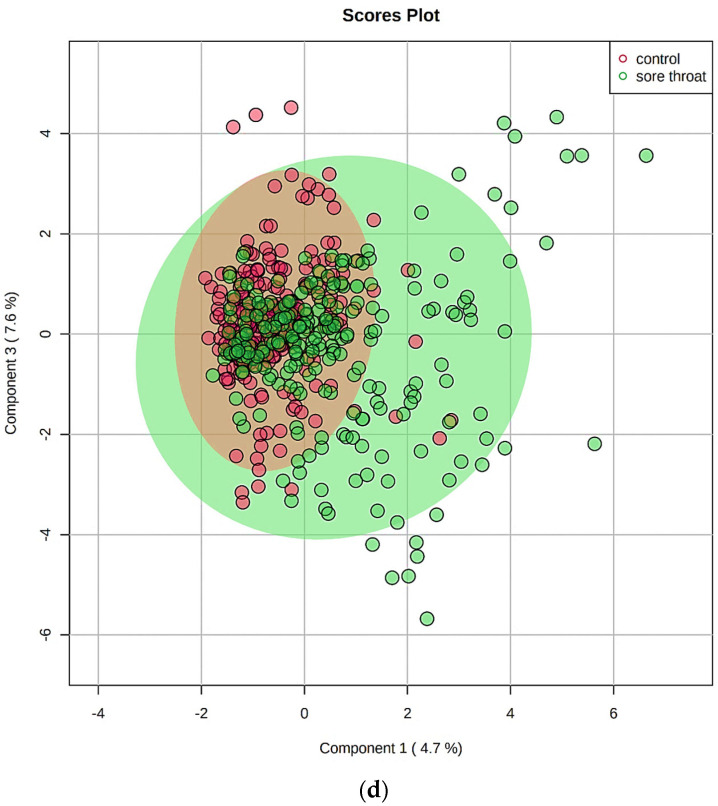
(**a**,**b**) 3D component 3 vs. component 2 vs. component 1 PLS-DA scores plots of the TSP-normalised ^1^H NMR WMS supernatant dataset for exploring distinctions between participants with an acute sore throat condition (green crosses) and those in an age-matched healthy control cohort (red triangles); in total, 240 samples from each group were analysed, i.e., 5 sequentially collected daily samples from each of 48 participants per group. A total of 31 assigned and named biomolecule predictor variables were incorporated into this experimental design (Model 1). In (**a**), row-wise product quotient normalisation (PQN), with glog-transformation and column-wise Pareto-scaling were applied prior to analysis; (**b**) as (**a**), but with constant sum normalisation (CSN) applied in place of PQN. The % variance contributions for each PC are indicated for each model. (**c**) as (**a**), but with 183 ISB variables. (**d**) 2D component 3 vs. component 1 sPLS-DA scores plot of the complete 183 ISB variable ^1^H NMR dataset for a model featuring 10 ISB predictor variables per component for a maximum number of 4 components (PQN, glog-transformation and Pareto-scaling were all applied to the MV dataset prior to analysis). This sPLS-DA analysis provided a high level of evidence for two separate sub-clusters of the sore throat group, one separate from the healthy control classification, the other appearing to co-cluster with it.

Additionally, an RF analysis performed on this TSP-normalised only dataset showed only 75.4 and 63.3% successful classification rates for the healthy control and sore throat disease-positive groups (Figure 5), and this poor classification success rate may arise from the sub-clustering of some sore throat group participants within the control group, as is clearly shown in the sPLS-DA scores plot displayed in Figure 6 below. However, these values were not unexpected in any case in view of the poorly discriminating PLS-DA results acquired.

Subsequently, further analysis was performed as above, but with the application of PQN normalisation, which utilised a mean metabolite (or ISB) level reference profile for the healthy control group. This approach was employed for the purpose of removing any WMSS concentration problems arising from dehydration/SFR issues in the sore throat participant cohort. Notwithstanding, this analysis also found only a marginal level of discrimination between these two clinical group classifications (Figure 6a). Indeed, it also generated a Q^2^ value of only 0.19, although again there was a very highly significant permutation test *p* value of ≤5.0 × 10^−4^. The order of important metabolite variables found, i.e., those with VIPs of ≥0.90, was glutamate (↑) > glutamine (↓) > pyruvate (↓) > urea (↑) > 3-D-hydroxybutyrate (↓) > alanine (↓) > taurine (↓) > N-acetylsugars (↑) > choline (↓) > formate (↑) > phenylalanine (↓) > succinate (↓). Again, the top four of these significant predictor variables, along with N-acetyl transfer metabolites and succinate, again are mainly derived from the host and not the oral microbiome source. Furthermore, the corresponding RF model performed showed 73.3 and 71.3% successful classification rates for the healthy control and sore throat disease-positive groups, and although this was higher for the latter than that observed in the previous non-PQN-normalised dataset, this somewhat limited classification success rate may again arise from sub-clustering of some ST group participants within the control group, as shown throughout Figure 6, but most notably in Figure 6d. However, as found above, these values were not expected to be very high in any case in view of the above PLS-DA results acquired.

Hence, the majority of key distinguishing biomarkers detected appear to arise from the host source (along with some contributions from the oral microbiome-based component 2, as noted in Figure 6), and these mainly account for the at least partial distinction between the control and acute sore throat disease classifications. However, on reference to Figure 6a–c, it appears that some augmentation of this cluster separation is also mediated by the as yet largely undefined source of component 3; strictly, the PQN normalisation process should be employed for these assessments, since this was the case on our original factor analysis reported in Ref. [1]. From analysis of this PQN-normalised dataset, metabolites loading significantly on this component included a series of aromatic amino acids, and methylamine. Using a significance analysis of metabolomics (SAM) approach (with Delta and false discovery rate (FDR) control values of 0.30 and 0.038, respectively, and assuming equivalent intra-ISB variable variances for each group for comparison), methylamine was discovered to be present at significantly higher concentrations in the sore throat disease group (*q* = 0.051). Indeed, this malodorous amine had a fold-change value of 1.52, whereas that for trimethylamine was 1.62 (in addition to PQN normalisation, this dataset was also glog-transformed and Pareto-scaled prior to analysis).

A further repeat of this analysis, but this time applying CSN to the Model 1 nominated metabolite dataset in place of PQN, which was also utilised for the circumvention of any modifications in WMSS metabolite levels derived from potentially diminished SFR values in the sore throat group. This analysis, however, also only achieved a low Q^2^ value (0.17), although it also had a very highly significant permutation test *p* value of <5 × 10^−4^ (3D scores plot shown in Figure 6b). Similarly, important variables found for this analysis were glutamine (↓) > pyruvate (↓) > glutamate (↑) > taurine (↓) > succinate (↓) > urea (↑) > thymine (↓). Again, the majority (7/8) of these efficiency-selected biomarkers were those arising from the host and not oral microbiome sources. The same RF model yielded 73.1 and 73.8% successful classification rates for the healthy and sore throat participant cohorts, i.e., the former rate was similar to that achieved with PQN, although it was improved very slightly for the latter classification. Likewise, performance of this Model 1 CSN dataset analysis on all 183 ISB variables yielded similar results, with Q^2^ = 0.15 and a permutation test *p* value < 5 × 10^−4^. Again, components 1 and 3 appeared to be largely responsible for the between-disease group discrimination found.

The sPLS-DA scores plot arising from the analysis of the full Model 1 ^1^H NMR dataset with 183 ISB variables, and featuring 10 ISB predictor variables per component for a maximum number of 4 components, is shown in Figure 6d (PQN, glog-transformation and Pareto-scaling were all applied to the dataset prior to analysis). This plot again provided a high level of evidence for two separate sub-clusters of the ST group, one separate from the healthy classification, the other appearing to co-cluster with it, an observation again perhaps indicating that a proportion of the ST participants did not actually have, or perhaps were only starting to develop, a sore throat condition. This separation of the sore throat group into two almost distinctive sub-clusters is also apparent for the PLS-DA scores plots shown in Figure 6b,c.

**Figure 7:**
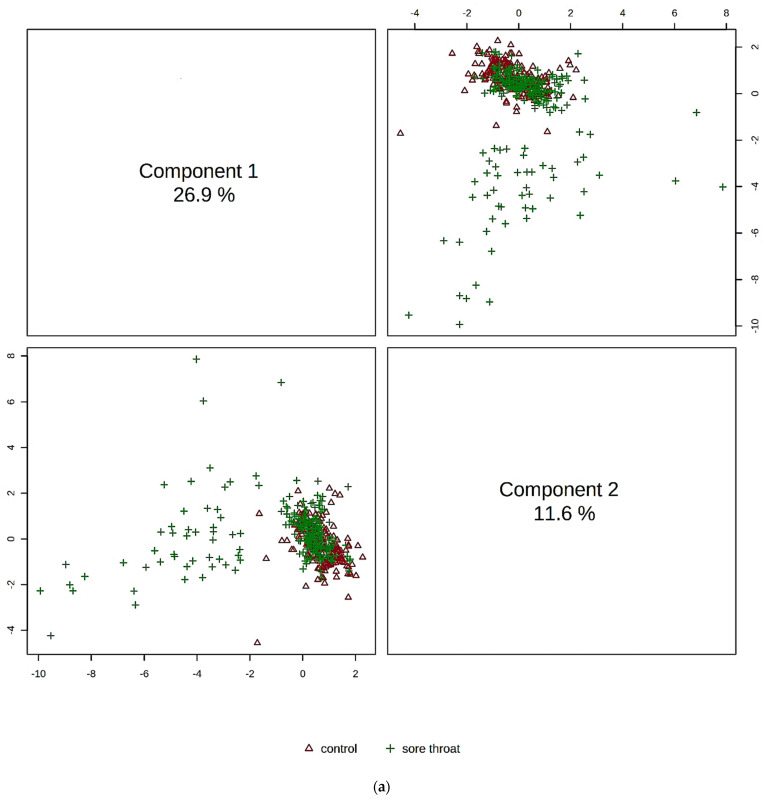

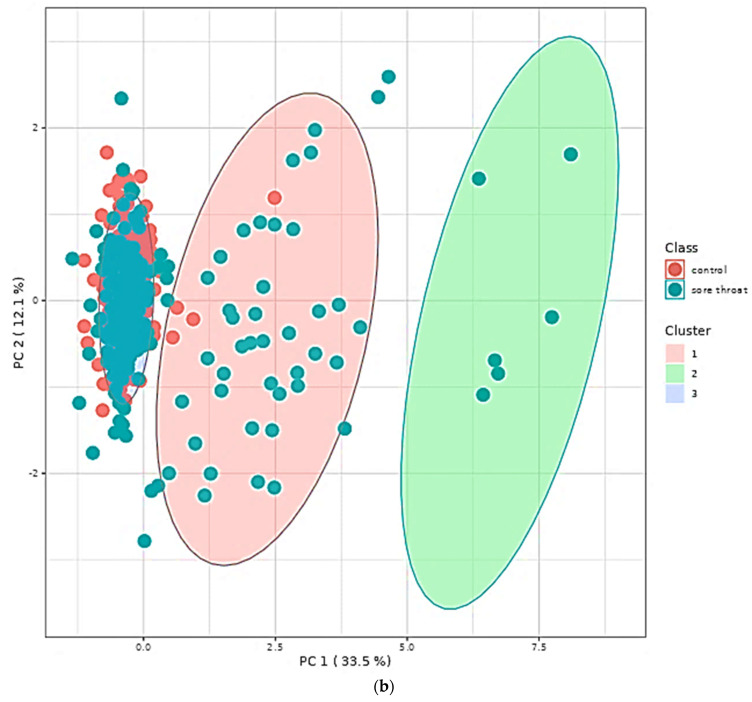
(**a**) Further segregation of the factor analysis (FA)-defined host source metabolite PC1 component (Ref. [1]) into two sub-components of 10 variables each via a secondary sPLS-DA strategy: PC2 versus PC1 and PC1 versus PC2 two-dimensional (2D) scores plots of results arising from this analysis of the acute sore throat disease dataset, with n = 480 participants (240 sore throat-positive, 240 age-matched healthy controls) and 31 ^1^H NMR-assigned biomolecules. Model 2 variance contributions for PC1 and PC2 were 26.9 and 11.2%, respectively. (**b**) PC2 versus PC1 2D scores plot of a composite PCA/k-means clustering analysis applied to the full Model 1 ^1^H NMR ISB dataset (183 variables in total). Estimated variance contributions for PC1 and PC2 were 33.5 and 12.1%, respectively.

An alternative approach to this MV sPLS-DA analysis was that featuring a Model 2 strategy, which involved segregation of the assigned, significant human host metabolite-derived variables (i.e., those loading on PC1 in Ref. [1]) into two components with 10 variables each, and with the same normalisation, glog-transformation and Pareto scaling approaches (Figure 7a). This approach also yielded a maximal Q^2^ value of 0.17 (permutation *p* value also <5 × 10^−4^), the important discriminatory variables with VIP values >0.90 being glutamate (↑) > glutamine (↓) > pyruvate (↓) > urea (↑) > taurine (↓) > succinate (↓). RF analysis revealed marginally higher successful classification rates of 79.2 and 73.3% for the healthy and sore throat disease groups, than those found with a model involving all 31 assigned metabolites.

Additionally, a PCA strategy was applied in conjunction with a k-means clustering analysis in order to attain a quintessential clustering of this acute ST study dataset. This procedure involved (1) dimensionality reduction through use of the former technique, and (2) the fitting of PCA-derived PCs to the k-means algorithm (rather than original ‘predictor’ variables) in order to determine the optimal number of dataset clusters. These clustering results were achieved via measurements of the sum of the squared distances of PC scores to their nearest cluster centroids. For all solutions, the within-cluster sum-of-squares values were then computed, and from these, together with employment of the % variance accounted for versus PC number ‘elbow’ plot approach, a computerised decision regarding the number of clusters to be retained was reached. This composite MV analysis approach found that the best model was that with three such clusters, as shown in Figure 7b. These clusters were segregated in the left-hand side, centre and far right-hand side sectors of the scores plot PC1 axis (centroidal PC1 values of these were ca. −0.5, 2.4 and 6.7, respectively). The central and right-hand side clusters (clusters 1 and 2) both contained only sore throat-positive participants, whereas the tighter but more populated left-hand side cluster (cluster 3) contained a sample-intensive admixture of both healthy control and sore throat disease participants. Hence, this form of analysis also indicates that at least some of the apparently sore throat-positive individuals may have been incorrectly selected or diagnosed on the basis of screening information which they provided to researchers, and were therefore possibly healthy individuals, and classifiable as such in a MV metabolomics analysis context; however, it remains possible that some of these incorrectly defined participants were only just beginning to develop this condition at the time of sample donation. Of further interest, this type of combinatorial MV analysis appears to have further distinguished two sets of sore throat patients, although that of cluster 2 only contained n = 6 participants. Cluster 1 contained n = 44 WMSS samples from sore throat participants, but only four from healthy control ones.

In summary, the incorporation of all possible assigned resonance variables, along with that incorporating all possible ^1^H NMR ISB variables, only very marginally improved the discriminative capacity of each PLS-DA model applied. However, the RF classification success rate was found to be improved somewhat when including only those significantly loading on the host-derived metabolite factor analysis PC (PC1 in Ref. [1]). As with PLS-DA, the majority of the important variables selected by the all-metabolite variable model were those featured in the host source-loading variable only dataset.

Therefore, it appears that the acute sore throat condition in humans appears to mainly induce dysregulations in human host metabolic pathways, particularly those with significant to substantial impacts from two or more of the above host-derived metabolites. Particularly noteworthy was the observation that a quite high fraction of the key biomarker metabolites identified were those involved in various amino acid pathways, along with the aminosugar, glycolysis/gluconeogenesis, urea cycle, and nicotinate and nicotinamide routes, amongst others. Notwithstanding, full quantitative metabolite enrichment and pathway topological analyses of these (relative) concentration perturbations to human metabolic pathways are outlined in Section 5.6 below.

### 5.6. Quantitative Metabolite Enrichment and Pathway Topological Analyses

Quantitative metabolite set enrichment analysis (MSEA) was conducted with a *Homo sapiens* host system. This analysis was limited to metabolites classifying as host-derived ones only for two major reasons: Firstly, distinctions between the acute sore throat and healthy control participant groups largely involved differences between the salivary concentrations of such biomolecules, and secondly, to circumvent any complexities introduced by salivary microbiome metabolites, which clearly cannot be considered in a human metabolome context (as required, the *Homo sapiens* pathway library was selected for this and the pathway topological analyses conducted). Therefore, the metabolites featured in this analysis were restricted to choline, glutamate, glutamine, glycine, lactate, ‘free’ N-acetylsugars, N-acetylamino acids, pyruvate, succinate, taurine, thymine, tyrosine and urea only.

**Figure 8:**
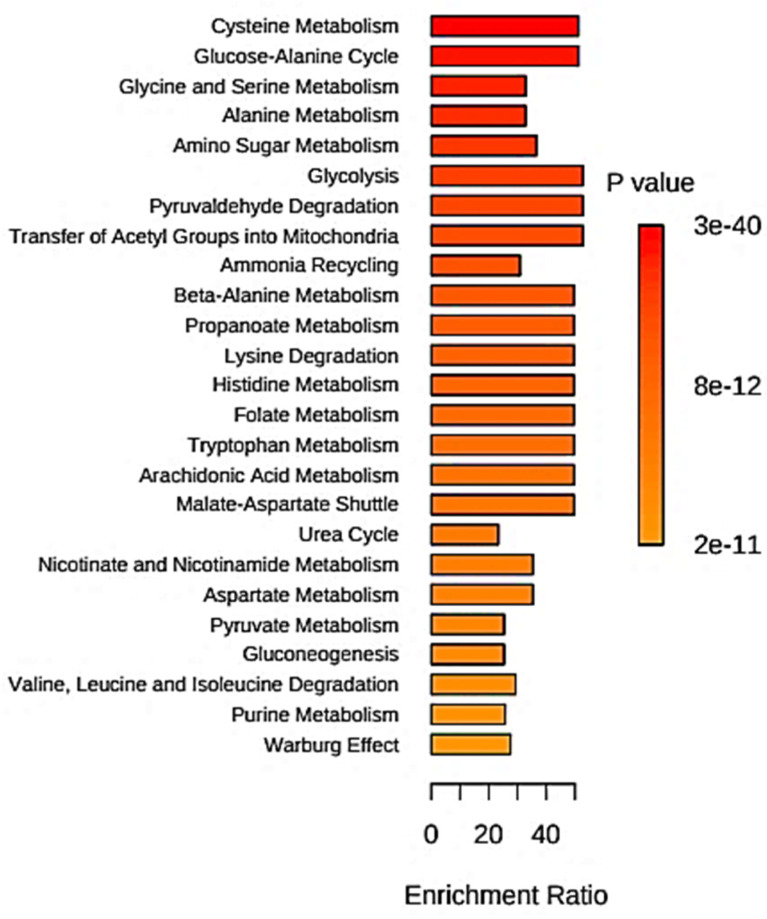
Overall summary plot for Quantitative Enrichment Analysis (QEA) performed on human host-contributory salivary metabolites, showing enrichment ratios and corresponding *p* values for the top 25 metabolic pathways which are indicated as being the most highly dysregulated for humans affected by an acute sore throat condition. QEA was performed using the *globaltest* package option [88], which employed a generalized linear model to estimate Q statistics for metabolite sets; such Q statistics represent correlations between metabolite concentration profiles (predictor X variables) and the clinical outcome (Y variable). The Q statistic provided for a metabolite set represents the mean value of the Q statistics for each metabolite within that set. The small molecule pathway database (SMPDB) metabolic pathway-associated metabolite sets consisted of 99 entries.

On completion, MSEA selected 25 human host metabolic pathways implicated in the pathogenesis of sore throat disease, with very high significance (*p*) values ranging from 10^−39^ to 10^−10^ (Figure 8). The six most significantly perturbed pathways were found to be cysteine metabolism, the glucose-alanine cycle, glycine and serine metabolism, alanine metabolism, aminosugar metabolism (also relevant to ^1^H NMR-detectable APP carbohydrate side-chain N-acetylsugar residues, along with their ‘free’ sugars), and glycolysis. Therefore, these imbalances appear to represent the key metabolic host responses involved in the human pathological response to acute sore throat conditions.

**Figure 9:**
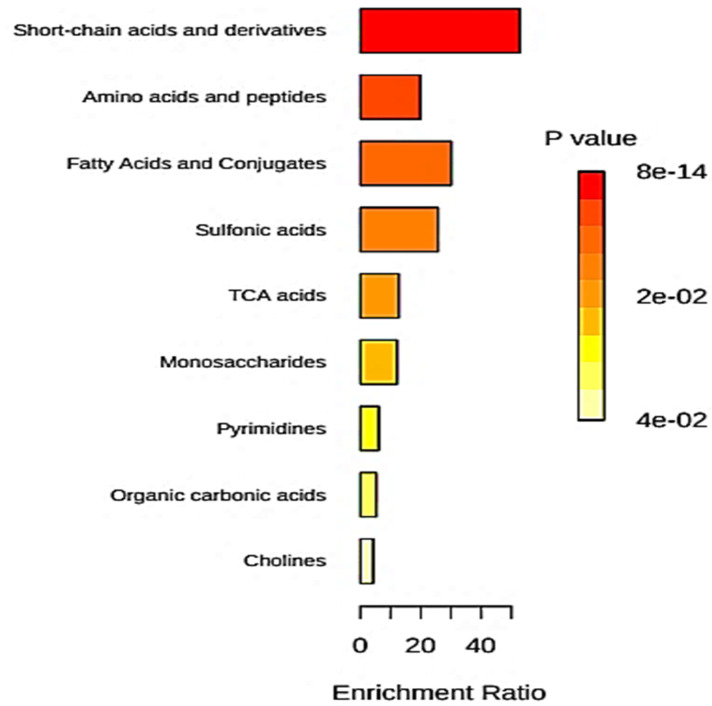
**Main Family Metabolites—Enrichment Overview.** Enrichment overview of FDR-corrected *p* values for main family metabolites: short-chain acids and derivatives, 7.08 × 10^−13^; amino acids and peptides, 2.78 × 10^−10^; FAs and conjugates, 6.78 × 10^−9^; sulphonic acids, 6.33 × 10^−7^; tricarboxylic acid cycle (TCA) acids, 5.99 × 10^−4^; monosaccharides, 6.79 × 10^−4^; pyrimidines, 1.75 × 10^−2^; organic carboxylic acids, 2.40 × 10^−2^; and cholines, 3.99 × 10^−2^. This analysis used the KEGG 80-entry pathway-analysis metabolite sets for *Homo sapiens*, and the computation of Q statistics was that described above in Figure 8.

A further enrichment overview provided FDR-corrected *p* values for a series of main metabolite families (Figure 9). Clearly, those for short-chain acids and derivatives, amino acids and peptides, free FAs and conjugates, sulphonic acids, tricarboxylic acid cycle (TCA) acids and monosaccharides were all very highly significant. These metabolite families are clearly relevant to the involvement of such classes of compounds in the significantly disturbed metabolic pathways specified in Figure 8, and those discovered in the pathway topological analysis described below (Table 2). For example, pyruvate and lactate are examples of short-chain organic acid anions (SCOAAs), which although were mainly PC1-loading and are involved in a range of host metabolic pathways, they are also well-known microbial catabolites; however, formate, a further SCOAA, which is involved in glyoxalate and dicarboxylic acid metabolism in humans as noted in Table 2 above [89], preferably loaded on another unsourced factor analysis component (PC4) from Ref. [1], together with histidine; cysteine, alanine and glutathione metabolism are routes featuring amino acids and peptides (the co-loadings of histidine with formate on this fourth PC is consistent with the role of this amino acid in one-carbon metabolism—it also serves as a key dietary precursor of formate [1,88]); arachidonic acid/arachidonate metabolism involves FAs and their conjugates; the β-amino acid taurine, which is featured in cysteine and methionine, and glutathione metabolism pathways, has a sulphonate functional group; succinate is a key metabolite in the citric acid cycle. Choline metabolism is closely inter-linked to methionine metabolism (along with that of folate), since all these pathways influence the generation of S-adenosylmethionine, which is the ubiquitous donor of methyl groups in biosystems [90]. Moreover, pyrimidine metabolism features urea, and feeds into the arginine biosynthesis, β-alanine metabolism and BCAA degradation pathways in humans, and has a bidirectional feed with the alanine, aspartate and glutamate metabolism pathway [89].

Although the highest pathway impact parameters were those estimated for phenylalanine, tyrosine and tryptophan biosynthesis (0.50), and taurine and hypotaurine metabolism (0.43) (not shown in Table 2), their corresponding FDR-corrected *p* values were 0.59 and 4.00 × 10^−7^, respectively, so the former cannot be viewed as significant. When considered overall, however, it should be noted that for virus infections in general, metabolic pathways featuring glucose, FAs and glutamine serve as key host metabolic routes by which viruses in general may exploitatively dysregulate [73], and here we found that glycolysis/gluconeogenesis, and alanine, aspartate and glutamate metabolism, were very highly significantly included in the list of ‘pharyngitis-disturbed’ pathways provided in Table 2. Furthermore, carbohydrate, along with lipid metabolism, are known to be significantly disturbed by HIV infection [76].

Arginine and proline metabolism represents one of the key pathways for the biosynthesis of arginine and proline from glutamate, the latter of which therefore plays an important precursorial role; routes linking these amino acids are bidirectional. Of the above biomarkers identified from the PLS and RF analysis strategies applied, alanine, pyruvate, glutamine and succinate are all involved in the alanine, aspartate and glutamate metabolism pathway. Cysteine and methionine metabolism, however, features pyruvate, taurine and glutamate, whereas glyoxalate and dicarboxylic acid metabolism involves glutamate, glutamine, acetate and formate. Histidine metabolism features glutamate and histidine itself. Glutamine, acetate and lactate are biomolecules engaged in pyruvate metabolism, and nitrogen metabolism involves formate, glutamine, glutamate and urea; in the latter overall major pathway, urea is generated as a non-toxic soluble metabolite for the removal of toxic ammonia arising from amino acid catabolism in the urea cycle. Butanoate metabolism involves acetoin, in addition to pyruvate and succinate (acetoin was identified as a biomarker in Ref. [74]), although pyruvate is, of course, the terminal end-product of the glycolysis pathway. Finally, glutathione metabolism involves glutamate, taurine and glycine.

Of notable interest, the alanine, aspartate and glutamate pathway feeds into the histidine, nitrogen, glutathione, butanoate, and glyoxalate and dicarboxylate metabolism routes, and this undoubtedly contributes towards the extremely highly significant FDR-adjusted *p* values reported in Table 2 and Figure 8, along with the very large participant and within-participant replicate sample sizes, which are highlighted in the experimental design of this study. Further ‘feed-in’ metabolic pathways from the alanine, aspartate and glutamate one includes the porphyrin, cyanoamino acid, C-5 branched dibasic acid and lysine biosynthesis routes.

## 6. Diagnostic Metabolomics Analysis of Saliva Samples Collected from Animals in Veterinary Studies

Critically, metabolomics analysis of saliva is not limited to humans alone, and very recently Sanchez et al. [91] applied this multianalyte technology to determine if panels of molecular markers of porcine health status available in saliva samples offer improvements over those usually monitored in blood serum, experiments aimed at detecting diseases in these animals in pilot field environments.

Following the application of detailed statistical analysis regimens, results acquired demonstrated that saliva could be used as an alternative for serum for acute-phase protein (APP) determinations in view of favourable agreements observed between these biofluid estimates. However, for all other potential biomarkers examined (C-reactive protein (CRP), the inflammatory markers haptoglobin (Hp), adenosine deaminase (ADA), total antioxidant capacity (TAC), and total protein content (TP), along with the concentrations of the essential trace elements copper and zinc ions), no such agreement was found. Notwithstanding, salivary ADA and TP levels were significantly greater in diseased animals, although health distinctions using their serum concentrations only were inconclusive. Additionally, a higher level of discrimination between healthy and diseased animals was observable when the overall MV distribution of these biomarkers were analysed in saliva, but not in corresponding serum media. However, in future, appropriate regression models may serve to provide an optimal signature of useful biomarkers for disease diagnosis in porcine saliva (Hp, CRP, and TAC) and/or serum (Hp, CRP and total copper ions), a selective method which may be less laborious and costly for determinations made on this biofluid. Hence, salivary biomarkers instead of those in blood serum may provide a more efficient means for the identification of diseased animals. An updated review of differences between the patterns of AAP and lower saccharide resonances in the acetamido-NHCOCH_3_ regions of the ^1^H NMR profiles of human saliva and blood plasma is outlined on Part II of this series of reports based on salivary metabolomics and their diagnostic/prognostic monitoring potential. Results acquired from further experiments focussed on determinations of the molecular structures and sources of these potential markers, for example host or exogenous food sources, particularly for potential low-molecular-mass markers such as N-acetylneuraminate, will be reported elsewhere.

Similarly, Turunen et al. [92] explored the canine salivary metabolome in order to prospectively monitor molecular mechanisms associated with the pathophysiological status of dogs (*Canis lupus familiaris*). Such developments are, of course, invaluable for the probing of canine diseases and conditions in veterinary medicine. In this study, stimulated saliva specimens were collected from cohorts of privately-owned dogs and human participants (n = 13 and 14, respectively), and untargeted ultra-high-performance liquid chromatography-quadrupole time-of-flight mass spectrometry (UHPLC-qTOF-MS) was employed to determine the biomolecular profiles of these biofluids. Results obtained revealed that of the total of 211 endogenous and exogenous salivary metabolites quantified, 25 lipidic agents were found in dog saliva which were absent from that of humans, and eight dipeptides were detected only in human and not dog saliva. Hence, despite a large variation in ion abundance, the metabolic profile of dog saliva was distinct from that of humans. Hence, biomolecules determined in this investigation suggested that canine saliva is prospectively a versatile matrix for the exploration of biomarkers applicable to the monitoring of dog welfare and health status. However, further studies are required to further confirm and validate these results.

Feline odontoclastic resorptive lesion (FORL) represents a quite frequent oral condition in cats, and its incidence has been promoted via the domestication of these animals. However, to date, although the aetiology of this disease has not been resolved and verified, cat feeding regimens, together with vaccination, and neutering processes, have all been implicated in the pathogenesis of FORL disease. Therefore, Ramadan et al. [93] evaluated the practicability of ^1^H NMR- and LC/MS-linked metabolomics strategies to diagnose this condition at its early stage onset, so that associated diagnostic biomarkers found could be employed for the purpose of interventions for curtailing or reducing the adverse progression of FORL. For this purpose, saliva samples collected from groups of 11 healthy and 10 FORL diseased felines were analysed by the above techniques, and clear differences between their biomolecular profiles were found (cross-validated PLS-DA was employed to determine this distinction). However, the model obtained was only able to predict FORL-disease in cats with only >60% accuracy. Moreover, the maximal PLS-DA Q^2^ value obtained was <0.30. However, cats with FORL demonstrated enhanced concentrations of a wide range of organic acid anions and amino acids, including acetate, isovalerate, lactate, propionate, phenylalanine and tryptamine, and these changes, according to the authors, suggested modifications to the oral microflora in FORL disease. However, our recent studies have found that it is possible to segregate factor analysis components attributable to the oral microbiome and host sources, with many selected organic acid anion (e.g., propionate) and fewer amino acid catabolites arising from the former (catabolites from both saccharolytic and proteolytic bacteria, respectively). A range of further metabolites, including other amino acids, lactate, N-acetylsugars and APPs, could also be sourced in this manner. Overall, the authors of Ref. [93] concluded that their investigation was only preliminary, and that a study with a larger feline cohort providing more samples would be required to supplement validation of the above biomarker profile for predicting the pathophysiological status of, and metabolite pattern imbalances in, FORL disease.

## 7. Discussion Featuring (1) Considerations of Salivary Biomarker Validation; (2) Prospective Applications of Metabolomics Data to ‘Real-Life’ Clinical Diagnoses and Disease Severity Monitoring; and (3) Potential Global Applications of the Techniques Developed

### 7.1. Recommended Strategies for the Validation of Salivary Biomarkers Discovered in Metabolomics Studies

For ‘omics’ investigations utilising metabolomics biotechnologies for the identification, stratification and monitoring of human diseases using WMSS samples as a biomarker source, it is essential to apply rigorous scientific and clinical validation protocols prior to their adoption for clinical screening programmes. Such validation programmes should ideally be supported by the involvement of standard operating procedures (SOPs) for testing regimens performed with this biofluid. Notably, optimal biomarker screening strategies should involve amalgamations of both current and newly developed bioanalytical techniques.

#### 7.1.1. Bioanalytical and Statistical Validation of Biomarkers (Internal and External)

Validation represents ‘a process to establish that the performance of a test, tool, or instrument is acceptable for its intended purpose’ [94,95]. Primarily, internal validation serves to determine the performance of a biomarker within the dataset in which it was first discovered, and should primarily be evaluated by extensive statistical resampling strategies, including bootstrapping or cross-validation (for example, leave-one-out (LOO) cross-validation, etc.), in order to provide realistic and acceptable presuppositions [96,97]. However, external validation serves to confirm the performance of a biomarker when present in a wholly independent dataset, and which is not exploited during its development; indeed, it must be independently identified and provisionally tested in datasets collected from differing and distinct testing sites and geographic areas, and at different time periods and perhaps durations.

Bioanalytical and clinical validation strategies represent two distinguishable phases for the validation of biomarkers. Indeed, in many protocols, the analysis of samples prospectively collected from target populations (both healthy control and disease study groups) prior to the acquisition of information on participant/patient outcomes serves as an important aspect of experimental design, and which is applicable to all such validation protocols, and for which the circumvention of experimental bias is critical [96].

Primarily, the major objective of bioanalytical validation is the enactment and institution of the performance characteristics and metrics of single biomarkers, or alternatively distinctive patterns of more than one of them, through evaluations of the specificity, sensitivity, accuracy, precision, and inter-laboratory or -institutional analytical reproducibilities. Any further recognised and relevant performance characteristics should also be included. Subsequently, a series of MV statistical analysis assessments are utilised for analytical validation; these approaches are not dissimilar to those applied in biomarker discovery and include the generation of suitable receiver operating characteristic (ROC) curve plots of true positive prediction rate (sensitivity) against false positive rate (1-specificity); discrimination, i.e., how effective is/are the biomarker(s) concerned at discriminating disease cases from demographically matched healthy controls; and calibration, specifically how efficacious is the model established to estimate the disease risk or other criteria? Overall, the major role of analytical validation is to determine the technical reliability of biomarker(s) to provide consistent estimates of disease or disease activity when evaluated against previously unknown parameters.

#### 7.1.2. Clinical Validation of Biomarkers

The major objective of clinical validation is to provide an acceptable correlation between the biomarker(s) and the pre-specified clinical output end-point, and often also to determine the clinical usefulness and validity of the measures proposed [98]. Clinical validation principally depends on an external validation process, and may be conducted either by the retrospective assessment of already available clinical trial datasets, and/or by the design and operation of new prospective clinical trials. Notably, the retrospective utility of pre-existing clinical trial datasets usually represents a form of external clinical validation for which the exploration of biomarkers was not considered to be an aim of the original experimental design.

However, in general, the establishment of clinical biomarker application, or clinical usefulness, will certainly require a new prospective clinical trial as a form of external validation, most especially to provide reliable evidence for value of the biomarker(s) evaluated, and to determine whether their use can give rise to improved health outcomes, if any. Readers are therefore referred to one very good example of this process, which is the US Food and Drug Administration’s (FDA’s) approval of pembrolizumab [99,100], a humanised antibody treatment for cancer immunotherapy, and which has valuable uses as a flagship therapy for a range of cancer conditions, including melanoma and Hodgkin’s lymphoma.

Of the available study designs for biomarker validation, target populations for the biomarker specified may indeed already be available in salivary sample biobanks which have been prospectively donated [101]. In such cases, what is known as a prospective-specimen-collection, retrospective-blinded-evaluation design [102] can be conducted in order to validate the screening of diagnostic biomarkers, along with those to be applied for prognostic monitoring purposes. Primarily, both samples and associated clinical data should be collected without any knowledge regarding the disease status of participants/patients and/or their clinical outcome(s). Random selection of both control and disease case participants should be applied on consideration of their outcome status, and then biomarker datasets are produced for each participant selected, albeit with investigators fully blinded to all clinical and related outcome results.

In summary, it is thoroughly recommended that any key diagnostic biomarkers determined from NMR-linked salivary metabolomics investigations should only be employed for the diagnosis and/or prospective prognostic implications of human diseases when correctly and reliably validated, specifically through the above bioanalytical, statistical and clinical routes, the latter perhaps representing the most important of these. Indeed, from the clinical standpoint, a full acceptance of these validation strategies may only be accomplished when it has been proven that the biomarker(s) concerned respond in a correlative fashion to treatment with known drugs which already have a fully proven therapeutic record as a therapy for the disease process in question. However, unfortunately, this is not a strategy which has been adopted by very many metabolomics researchers whose pivotal focal point is the seeking, identification and quantification of biomarkers in study biofluid samples. Moreover, the authors of the current study also advocate that all ‘state-of-the-art’ metabolomics biotechnologies employed, preferably those involving more than just a single bioanalytical technique, should be employed concurrently with more conventional and pre-established approaches for disease diagnosis and monitoring. For example, supporting associated clinical and microscopic examinations, along with histopathological gradings, where relevant, should also be employed to permit evaluations of any significant associations between these two types of diagnostic monitoring episodes. Once proven, then in principle the ‘omics’-based analysis of WMSS or other oral fluid samples may be employed for independent clinical monitoring episodes.

### 7.2. Translation of Biomarkers Discovered by Metabolomics Techniques to ‘Real-Life’ Clinical Diagnoses and Monitoring of Human Diseases

Overall, it remains a rather regrettable fact that many of the unique and novel developments made in the salivary metabolomics research area for the simultaneous multi-analyte evaluation of this biofluid for diseases or disease activities have not readily been accepted and adopted for routine clinical screening purposes at hospital or clinical laboratory sites, for example. As already noted above, one major problem lies with the only very limited attempts made for the full validation of biomarkers determined, both bioanalytically/statistically and clinically based. A further drawback is the bulky frameworks and sometimes very high purchase and running costs associated with the facilities required for these purposes, for example liquid or gas chromatographic/mass-spectrometric (LC or GC/MS)-, inductively-coupled plasma (ICP)/MS. or matrix-assisted laser desorption/ionization-time of flight (MALDI-TOF) MS techniques, in addition to high-resolution NMR facilities. Furthermore, all these techniques have a stringent requirement for specialist technical staff in order to operate successfully. However, one clear advantage is the selection and adaption of an array of perhaps five or so biomarkers with distinctive WMSS concentration ratio signatures which are characteristic of the disease in question; in principle, such patterns can be determined, potentially simultaneously, by relatively simple ELISA, biosensor and/or non-stationary point-of-service (POS) [103,104] methods targeted on disease diagnosis and/or its prognostic stratification.

Another alternative, very recently developed approach involves the future application of newly developed low-field (60–100 MHz), near-portable benchtop NMR spectrometers, which are cryogen-free, far less expensive, and very simple to operate and manage, even by previously untrained staff [1,105]. Such ‘state-of-the-art’ devices are easily installed and utilisable at patient ‘point-of-contact’ sites, e.g., local general practice and hospital clinics, dental surgeries, and community pharmacies, etc. However, although 12 or more metabolites are detectable in WMSS samples using this technique, currently limits of quantification dictate that only five of these (acetate, formate, propionate, glycine and methanol) may be determined in this biofluid when applied at an operating frequency of 60 MHz [105,106]. Indeed, for this biofluid, currently LF NMR spectroscopy is limited to metabolites with the most prominent signals, specifically those with clear singlets or simple first-order coupling patterns located within relatively uncrowded spectral regions. Nevertheless, soon this novel approach may have the capacity to more fully determine the metabolic status of WMSS samples, and hence offer potential for the routine screening of oral health conditions such as PD and dental caries, and prospectively also for extra-oral diseases.

A series of commercially available salivary testing systems or devices are currently available globally to selectively screen for, or provide valuable diagnostic information regarding, the nature or status of some clinical conditions [107,108]. Additionally included are determinations of salivary hormones and ethanol, together with drugs of abuse, Additional headway in the field of saliva-based biosensors has involved their non-invasive monitoring of glucose, lactate, phosphate, α-amylase and antibodies, together with selected cancer biomarkers [109]. Therefore, it may be conjectured that high-resolution NMR analysis, along with some other multi-analytical techniques, have provided at least some contribution towards the development of these important diagnostic monitoring devices, primarily to identify and then validate any relevant biomarkers for these purposes.

Further developments in this area include the detection and quantification of salivary lactate with an electrochemiluminescence cloth-based biosensor coupled with smartphone-linked imaging [110]; Lab-on-a-Chip lateral flow assays for hormone detection and monitoring [111]; an exploration of illicit drug use amongst ‘Boda Boda’ motorcyclists in Uganda, for which the investigators employed an ‘on-the-spot’ saliva drug test kit for a cohort of 785 participants [112]; and the applications of a series of microfluidic point-of-care (POC) devices in early diagnosis (portable inexpensive devices for a range of diseases, including cardiovascular disease detection) [103].

### 7.3. Comparisons of the Salivary Microbiome and Metabolome to Those to Those of the Human Gut: Potential Global Applications of Developed Salivary Metabolomics Techniques

Although the microbiomes of the oral and gut environments have long been known to have distinctive compositions, recent developments have now disclosed that bacterial strains from the former do colonize the latter much more extensively than previously conjectured [113]. Indeed, previously it was generally accepted that oral microbes only penetrate the gut environment in patients with particular disease conditions, for example colorectal cancer, inflammatory bowel diseases and rheumatoid arthritis [114]. Moreover, the stomach and bile acids in healthy humans were believed to diminish the penetration of bacteria from oral sources, so that significant levels of colonization therein are retarded [115].

Intriguingly, the now recognised extensive transmission of oral bacteria to the gut destination is of much physiological significance. Indeed, the wide array of low-molecular-mass organic acid anions (otherwise known as short chain fatty acids) found in human saliva, and which represent oral microbiome catabolites, largely have the same identities as those arising from anaerobic fermentation by microbial colonies available in the mammalian gut. Notably, total levels as high as 50–200 mmol./L may be generated in the human large intestine. Gut mucosa readily take up these catabolites where they may exert important actions as energy sources, and as receptor-recognisable signalling biomolecules, and mediators of gene expression [116,117]. Moreover, some quite recent developments involving the discovery of novel mechanisms for the abilities of organic acid anions to modulate immune cell development and counter inflammatory processes have also been delineated [118,119].

However, it certainly appears that the physiological effects of these agents are critically dependent on their precise molecular structures, with acetate attaining the highest systemic circulating levels, propionate facilitating liver gluconeogenesis, and *n*-butyrate being utilised as a gut mucosal energy font [116]. Moreover, as might be expected, there are some important contrasts between their interactions and equilibria with many proteins and protein receptors, for example, with reference to the abilities of propionate and *n*-butyrate to inhibit histone deacylases [120]. In view of these considerations, the microbial catabolic sources of these organic acid anions in the human gut, and hence prospectively also those in the oral environment, is of much importance, as is the possibility of their generation rates and levels to be influenced by modifications in either diet or gut physiology. The production of propionate and *n*-butyrate by human colonic mucosa was recently investigated by Louis and Flint [121], and this study confirmed that these two bacterial catabolites can indeed provide some favourable health benefits, for example facilitation of satiety and decreases in cholesterol intake for propionate, and defences offered against colorectal cancer for *n*-butyrate [119,122,123].

Notably, some of these agents may also arise from the attack of proteolytic bacterial enzymes on host proteins [56]. Nonetheless, a full consideration of the generation and metabolism of these compounds by differential colonies of gut and oral microflora is required in order to fully assess their potential benefits to the host system. Of special relevance to the current study, many, if not all, of the organic acid anions are readily detectable and quantifiable in the ^1^H NMR profiles of human saliva [3].

However, despite recent ‘state-of-the-art’ advances and developments in the field of molecular biology, which offers some valuable knowledge concerning our comprehension of the human oral microbiome, currently some key information related to its biomolecular and metabolic activities remain circumscribed [124,125]. Interestingly, saccharolytic bacteria therein (e.g., *Lactobacillus*, *Actinomyces* and *Streptococcus*) play important roles in the metabolic degradation of ingested carbohydrates to organic acid anions via the Embden-Meyerhof-Parnas glycolytic pathway and its linked branches. Such catabolites, particularly those in their protonated (acidic) forms, are involved in demineralisation processes associated with dental caries [22]. Since virtually all these species, together with a wealth of further relevant bacterial catabolites, and often their precursors, are readily detectable and quantifiable in metabolomics experiments as documented here, they all may act as relevant biomarkers for this and potentially other oral health conditions [1,2,125].

Intriguingly, the contents of the human gut, its microbiome, and metabolites/microbial catabolites therein are found to be strikingly well correlated with ethnicity and food habits, together with a series of environmental factors. For example, Wu [126] investigated the relationships of the gut microbiome and its metabolome to health and diseases, and found that the generation of selected bacterial catabolites is restricted by gut microbial composition; this evidence was achieved through comparisons of dietary intake, the gut microbiota, and the plasma metabolome between omnivores and vegans. Hence, these data indicated that dietary stratagems effectively mediate the gut microbiota and its metabolome, and that this approach could serve as a means for the maintenance of health, along with certain disease treatments. Moreover, Vojinovic et al. [127] explored the association between gut microbiota and circulating metabolites in a number of population-linked cohorts, and discovered a relationship of no less than 32 microbial families and genera, with circulating sub-fractions featuring very-low-density- and high-density-lipoproteins, serum lipids, ketone bodies, amino acids, ‘acute-phase’ glycoprotein markers, and glycolysis-related metabolites (gut microbiota have been previously associated with circulating concentrations of triacylglycerols and high-density-lipoprotein [127]). Therefore, these results provided evidence for the significant actions of gut microbiota in host metabolism. They also indicated that the gut microbiota may act as a prime target for suitable treatments or preventative measures.

Although the salivary microbiome has been associated with both oral and non-oral diseases, until recently there was little information available on the influence of various environmental variables on this factor and the salivary metabolome. Such environmental factors include prolonged or lifetime dietary habits available. In 2014, De Filippis et al. [128] explored the effects of long-term omnivorous, ovo-lacto-vegetarian and vegan dietary options in human subjects on their salivary microbiota and metabolomes. In this study, the microbial diversity and biomolecular profiles of saliva specimens collected from a total of 161 healthy participants following these dietary regimens was investigated. In more than 98% of participants, a large microbiota fundament was found, and this featured 12 bacterial genera; these were sub-dividable into three classes of saliva sample types, which could be distinguished on the basis of their relative abundancies of some key genera, specifically *Prevotella*, *Streptococcus*/*Gemella* and *Fusobacterium*/*Neisseria*. However, statistical analysis suggested that there was no influence of dietary habit group on the salivary microbiota; moreover, phylogenetic beta-diversity analysis also unfailingly demonstrated that there were no significant differences between these groups of participants. Nevertheless, in contrast, metabolomics profiling of the saliva samples collected performed by both ^1^H NMR and GC-MS/SPME techniques was successful in the identification of diet-associated biomarkers which were significantly distinct between these dietary habit groups. Indeed, the salivary metabolites formate, 5-methyl-3-hexanone, urate and uridine were effective in distinguishing omnivore samples, and caproic acid, 1-propan-1-ol and proline were found to be representative of non-omnivorous dietary habits. Therefore, although salivary metabolites may form patterns which are characteristic of diet type, it appears that the oral microbiota itself is markedly resilient, and is not affected by differential dietary choices. Although microbial homeostasis may be disturbed by poor oral hygiene habits or alternative environmental contributors, this study concluded that there was no evidence available to suggest that any of the above dietary habits gave rise to differences in the oral microbiota’s compositional status, with sequelae influencing oral homeostasis.

In view of these significant environmental contributions towards the gut microbiota and metabolome, and the salivary/oral metabolome(s), it is, at least in principle, feasible that the field of salivary metabolomics in general may be viable for the investigation of differential disease processes on a global basis, with full considerations of background influences on participant or patient cohorts, which include their distinctive dietary habits and ethnicities, amongst other contributory factors.

## 8. Concluding Remarks

In this third part of our discourse focused on the applications of NMR-based metabolomics analysis of human oral fluids, particularly WMSSs, we have explored and critically reviewed a wide range of previous investigations focused on the diagnosis and prognostic stratification of both oral and largely orally systemic human diseases. We also present many of the important challenges that researchers working in this field are systematically faced with. Fortunately, not all these problems are insurmountable, and the information provided in this work hopefully provides some effective solutions to these. Indeed, in Section 3, we have reassessed the applications of such bioanalytical strategies to the oral health conditions PDs and dental caries, whereas in Section 4, we examined and re-evaluated a wider range of non-oral (systemic) diseases, notably types 1 and 2 diabetes, cardiovascular diseases, Sjögren’s syndrome, neurological conditions such as Parkinson’s and Alzheimer’s diseases, and finally viral diseases, including influenza, acute sore throat conditions, HIV and COVID-19 infections. From these studies, particularly notable were differences between participant instructions, WMS sample collection and its timing (particularly the inclusion, or not, of a sufficient fasting abstention period prior to this event), and sample preparation protocols. Moreover, for NMR-based investigations, the rigorous checking and validation of biomolecule spectral assignments, the heterogeneity of differential spectral acquisition parameters employed (including the use, or not, of water presaturation pulse sequences and those for suppressing the intensities of potentially interfering macromolecule resonances) and their influence on the intensities of metabolite signals in the profiles obtained, and the critical consideration of bioanalytical sensitivity, and hence the detectability and quantification of possible biomarkers, represent further important factors. Full outlines of the limitations of NMR- and non-NMR-based metabolomics investigations of human saliva conducted for the provision of chemopathological and diagnostic information are provided in Parts I and II of this series of publications (Refs. [1,2], respectively).

One systematic review focused on both periodontal diseases and oral cancer [62] found that the techniques and methods utilised for salivary metabolomics analysis, and the nature of disease-specific biomolecules detectable varied, and that a series of scientific challenges remain, notably an incomplete recognition of differential metabolic pathways involved in different disease states. However, this review provided an outline of the future potential opportunities offered by such strategies from a clinical perspective.

Further issues discussed herein included considerations of strategies employed for MV data analysis of metabolomics datasets and the ‘policing’ of these investigations; the potential employment of mouth-rinsed water washouts as ‘suitable’ alternatives to the analysis of WMSS samples; the effects of prior oral cleansing procedures on the metabolic profile of human saliva; and comparative evaluations of the biomolecular profiles of whole, parotid and submandibular/sublingual saliva samples.

The current study also explored the dataset normalisation preprocessing step, as discussed in Section 3.1.5 and in the case study presented in Section 5. It has been previously reported that the PQN approach, which originates from computation of the most plausible dilution factor by examining the quotient of the amplitude distributions of a real ^1^H NMR ‘test’ profiles and by expressing these relative to those of a pre-selected reference or average spectrum. However, no major ‘between-normalisation method’ differences in the classification success rates of each of the PLS-DA, sPLS-DA and RF analysis techniques applied to analyse our acute sore throat disease dataset, but introduction of the reportedly more robust and accurate PQN approach [29,129] would not, of course, necessarily be expected to achieve this.

Besides oral diseases, e.g., [130], salivary analysis also has a high level of potential applications for the diagnostics of various systemic diseases such as Sjörgen’s syndrome [65,66], as well as distant malignancies, such as breast cancer [131] and its recurrence [132], lung cancer [133,134] and prostate cancer [135] (recent developments in the diagnosis and prognostic monitoring of cancers are fully outlined and reviewed in Ref. [2]).

A further important clinical prospect is the possibility of oral-systemic health, i.e., the association of oral diseases with systemic health conditions. Indeed, the American Dental Association (ADA) has specified three key points for these linkages [136]: firstly, that PDs have been linked to other specified systemic conditions, such as diabetes and heart disease; secondly, that direct causality between these two disease classifications remains puzzling and therefore requires further clarification; and thirdly, that a large number of risk factors, for example poor diet and tobacco smoking, etc., are shared between them. Examples from the current study include those featuring associations between periodontal health and either diabetes [58], pulmonary disease [38] and oral cancer [129]. However, it is known that a very large number of systemic diseases and therapies available for their treatment impact on the status and health of the oral cavity, and that diseases in the mouth exert a more profound systemic impact than might be otherwise expected [137]. Indeed, in studies featuring high-field NMR analysis, this form of ‘spectral pathology’, along with its expansive upcoming prospects and future developments, should be thoroughly recommended for many future studies.

Some notable special considerations feature diseases involving virus infections. Since viruses essentially employ and remodel the metabolism of host cells, disease arising from human viral infections are particularly appropriate for contemporary ‘state-of-the-art’ metabolomics studies, NMR-based or otherwise, as noted here in our case study focused on the pathological and molecular mechanisms involved in acute sore throat conditions (Section 5). Indeed, the significant influence of viral activity on host metabolism during in vitro replication, and animal model or human infections, have to date contributed many novel mechanistic perceptions, and offered much potential for biomarker development and validation, and therefore drug targeting approaches. Of especial interest, the identification of metabolic pathways and pathway biomolecules used or adopted by viruses offers a range of opportunities for future anti-viral drug-targeting studies, and/or those targeted on investigating the actions of vaccines and their activities. Therefore, an improved understanding of the mechanisms by which viral species manipulate the endocrine system of the host, and multidimensionally interact with host proteins and enzymes, will facilitate the design and development of novel therapeutic regimens to circumvent and treat these infections.

Finally, of critical importance, the authors of the current study have also provided a quite expansive review of approaches available for biomarker validation, notably laboratory, statistical and clinical protocols for these, in Section 7.1.

## Figures and Tables

**Table 1:** Assignments of resonances in the 600 MHz ^1^H NMR spectra of WMS supernatant samples for the expanded δ = 0.80–2.45 and 5.10–8.60 ppm regions only. Chemical shift (ö) values and resonance coupling patterns are also provided. * Indicates tentative assignment. Resonances and their assignments within the 2.45–5.10 ppm regions of spectra (signals 47–95) are omitted for the purpose of clarity, but these may be viewed in Part I of this series of reports [1].

Assignment Code	Chemical Shift (δ/ppm)	Coupling Pattern	Assignment
1	0.861	*t*	*n*-Caproate-CH_3_
2	0.885	*d*	*iso*-Valerate-CH_3′_s
3	0.893	*t*	*n*-Butyrate-CH_3_
7	0.962	*t*	Leucine-CH_3_
8	0.982/0.996	2 × *d*	Valine-CH_3_/Isoleucine-CH_3_
10	1.035	*d*	Valine-CH_3_
11	1.058	*t*	Propioniate-CH_3_
12	1.125	*d*	*iso*-Butyrate-CH_3_
13	1.150	*d*	3-Amino-*iso*-butyrate-CH_3_
15	1.183	*t*	Ethanol-CH_3_
16	1.211	*d*	Methylmalonate-CH_3_/α-Fucose
17	1.242	*d*	3-D-hydroxybutyrate-CH_3_/β-Fucose
18	1.330	*d*	Lactate-CH_3_
19	1.371	*d*	Acetoin-CH_3_
20	1.486	*d*	Alanine-CH_3_
21	1.551	*q*	*n*-Butyrate-β-CH_2_
22	1.641	*m*	5-Aminovalerate-β,γ-CH_2′_s
Lys	1.71	*m*	Lysine-δ-CH_2_
25	1.76	*m*	Putrescine-β-CH_2_
26	1.77	*m*	Ornithine-β-CH_2_/Propane-1,3-diol-CH_2_-β-CH_2_
A_sat_	1.813/2.026	2 × *s*	Acetate-CH_3_ ^13^C satellite signals
β-NAcN-C3H_ax_	1.83	pseudo-*dd*	β-N-Acetylneuraminate-C3H position axial proton
30	1.92	*s*	Acetate-CH_3_
31	1.954	*m*	* 2-Hydroxyglutarate-γ-CH_2_
32	2.005	*m*	Proline-γ-CH_2_/N-Acetylneuraminate-C3H
33	2.02–2.080	*broad*	Glycoprotein-/Hyaluronate-/Glycoprotein carbohydrate side-chain N-acetylsugar-NHCOCH_3_ functions
34	2.025/2.030	*s*	N-Acetylglutamate-/N-Acetylaspartate-NHCOCH_3_ (2 signals)
β-Nac-CH_3_	2.041	*s*	β-N-Acetylglucosamine-NHCOCH_3_
β-NacN	2.06	*s*	β-N-Acetylneuraminate-NHCOCH_3_
37	2.098	*s*	Dimethylsulphide-S-CH_3_
38	2.140	*s*	Methionone-S-CH_3_
39	2.164	*t*	*n*-Butyrate-α-CH_2_
40	2.193	*q*	Propioniate-CH_2_
41	2.215	*s*	Acetone-CO-CH_3_
42	2.235	*t*	5-Aminovalerate-α-CH_2_
AcAc	2.27	*s*	Acetocaetate-CH_3_
2-OG	2.331	*t*	2-Oxoglutarate-4-CH_2_
43	2.334	*m*	Glutamate-γ-CH_2_
44	2.377	*s*	Pyruvate-CH_3_
Ox	2389	*s*	Oxaloacetate-CH_2_
46	2.415	*s*	Succinate-CH_2_
α-Fuc	5.209	*d*	α-Fucose-C1H
α-Glc	5.232	*d*	α-Glucose-C1H
UPH	5.24	*m*	Unassigned polyhroxy- species
96	5.392	*s*	Allantoin-CH
Sucr	5.414	*d*	Sucrose-C1H
CD-CH=CH	5.62	*m*	Conjugated diene species olefinic proton
97	5.67	*s*	Unassigned
98	5.79	*broad (s)*	Urea-CO-NH_2_
99	5.800	*d*	Uracil-C2H
Cinn	6.375	*d*	Cinnamate derivative-CH=CH-Ar
100	6.52	*s*	Fumarate-CH=CH-
101	6.84	*d*	4-Hydroxyphenylacaetate aromatic ring-C3H/C5H
102	6.85	*broad*	* Protein aromatic amino acid residue(s)
103	6.880	*d*	Tyrosine aromatic ring-C2H/C6H
104	7.071	*s*	Histidine imidazole ring-C5H
104A	7.155	*d*	4-hydroxyphenylacetate aromatic ring-C2H/C6H
105	7.237	*d*	Tyrosine aromatic ring-C3H/C5H
106	7.320	*m*	Phenylalanine aromatic Ring-C2H/C6H
107	7.375	*m*	Phenylalanine aromatic ring-C4H
108	7.43	*m*	Phenylalanine aromatic ring-C3H/C5H
109	7.533	*d*	Uracil-C1H
110	7.552	*m*	* Protein aromatic amino acid residue(s)
111	7.812	*s*	Histidine imidazole ring-C2H
112	7.913	*s*	3-Methylhisitidine imidazole ring-C2H
113	8.05	2 × *broad* signals	* Protein aromatic amino acid residue(s)
114	8.175	*s*	Hypoxanthine-C8H
115	8.219	*s*	Hypoxanthine-C3H
116	8.456	*s*	Formate-CH

**Table 2:** Pathway topological analysis showing the human metabolic pathways most significantly affected by acute sore throat disease pathology; the number of metabolites featured in each pathway, and the number of metabolite ‘hits’ are shown, as are the corresponding FDR-corrected *p* values for these contributions. The *Homo sapiens* pathway library was selected, which contained a total of 80 pathways. The chosen pathway library code was hsa (KEGG organisms abbreviation). The selected pathway enrichment analysis method and node importance measure for this toplogical analysis were *globaltest* and relative betweenness centrality, respectively. The ‘betweenness centrality’ determines the shortest path number passing through the node, and this is also employed as an importance measure for metabolites, since in this case the metabolic network is directed. The impact is the pathway impact value, which represents the matched metabolite node cumulative percentage values from all such nodes featured (computed from this pathway topological analysis). As many as 13 other metabolic pathways had FDR-corrected *p* values lying between 7.18 × 10^−11^ and 0.045, and these also may be implicated in the pathogenesis of pharyngitis, although less significantly so than those displayed in this Table.

Metabolic Pathway	Total Metabolites	No. of Hits	FDR	Impact
Arginine and proline metabolism	38	2	7.94 × 10^−39^	0.09
Alanine, aspartate and glutamate metabolism	28	4	3.38 × 10^−34^	0.31
Cysteine and methionine metabolism	33	1	6.94 × 10^−13^	0.00
Glyoxylate and dicarboxylate metabolism	32	4	6.94 × 10^−13^	0.11
Histidine metabolism	16	1	2.50 × 10^−12^	0.00
Nitrogen metabolism	6	2	1.99 × 10^−11^	0.00
Pyruvate metabolism	22	2	1.99 × 10^−11^	0.21
Glycolysis/Gluconeogenesis	26	2	1.99 × 10^−11^	0.10
Butanoate metabolism	15	2	2.27 × 10^−11^	0.00
Glutathione metabolism	28	2	7.18 × 10^−11^	0.10

## Data Availability

The data presented in this study are available in this article.
